# Supplementary material for: Differential in-hospital mortality and intensive care treatment over time: Informing hospital pathways for modelling COVID-19 in South Africa
Source: PLOS Glob Public Health. 2023 May 17;3(5):e0001073. doi: 10.1371/journal.pgph.0001073 (PMC10191304; doi:10.1371/journal.pgph.0001073)
Supplement: S1 Text — (DOCX) [file pgph.0001073.s001.docx]

# Supplementary material

**Table A**. **Province-specific time periods relative to the first three COVID-19 waves, defined by case report data (March 2020 to September 2021)**

|  | **Wave 1** | | **Wave 2** | | **Wave 3** | |
| --- | --- | --- | --- | --- | --- | --- |
| **Province** | **Start date** | **End date** | **Start date** | **End date** | **Start date** | **End date** |
| Eastern Cape | 01-Jun-20 | 12-Aug-20 | 20-Oct-20 | 25-Jan-21 | 10-May-21 | Data censor date (06-Sep-21) |
| Free State | 15-Jun-20 | 05-Nov-20 | 10-Dec-20 | 01-Mar-21 | 15-Apr-21 | Data censor date (06-Sep-21) |
| Gauteng | 01-Jun-20 | 24-Aug-20 | 28-Nov-20 | 05-Feb-21 | 01-May-21 | 15-Aug-21 |
| KwaZulu-Natal | 01-Jun-20 | 27-Aug-20 | 25-Nov-20 | 05-Feb-21 | 01-Jun-21 | Data censor date (06-Sep-21) |
| Limpopo | 15-Jun-20 | 06-Nov-20 | 15-Dec-20 | 01-Feb-21 | 20-May-21 | Data censor date (06-Sep-21) |
| Mpumalanga | 15-Jun-20 | 09-Sep-20 | 10-Dec-20 | 20-Feb-21 | 10-Jun-21 | Data censor date (06-Sep-21) |
| Northern Cape | 15-Jun-20 | 18-Nov-20 | 15-Dec-20 | 14-Apr-21 | 14-Apr-21 | Data censor date (06-Sep-21) |
| North West | 01-Jun-20 | 05-Nov-20 | 05-Dec-20 | 20-Feb-21 | 10-May-21 | Data censor date (06-Sep-21) |
| Western Cape | 01-May-20 | 28-Aug-20 | 10-Nov-20 | 01-Feb-21 | 15-May-21 | Data censor date (06-Sep-21) |

**Table B**. **Estimated probabilities of ICU admission, mechanical ventilation and death in the intensive care unit (ICU) and non-ICU for patients hospitalized for COVID-19, stratified by age, sector and time period across all provinces**

| **Age (years)** | **Time period** | **All patients** | **Non-ICU patients** | **ICU patients** | **Probability of ICU treatment** | **Probability of mechanical ventilation in ICU** | **Probability of death in non-ICU** | **Probability of death in ICU** |
| --- | --- | --- | --- | --- | --- | --- | --- | --- |
| **Public + Private Sector** | | | | | | | | |
| 0-14 | Wave 1 | 1,904 | 1,785 | 119 | 0.063 | 0.303 | 0.031 | 0.185 |
|  | Post-wave 1 | 724 | 685 | 39 | 0.054 | 0.333 | 0.018 | 0.077 |
|  | Wave 2 | 2,376 | 2,265 | 111 | 0.047 | 0.189 | 0.030 | 0.171 |
|  | Post-wave 2 | 1,172 | 1,124 | 48 | 0.041 | 0.333 | 0.028 | 0.042 |
|  | Wave 3 | 3,459 | 3,279 | 180 | 0.052 | 0.272 | 0.031 | 0.094 |
| 15-34 | Wave 1 | 13,064 | 12,510 | 554 | 0.042 | 0.339 | 0.042 | 0.273 |
|  | Post-wave 1 | 3,198 | 3,013 | 185 | 0.058 | 0.238 | 0.049 | 0.200 |
|  | Wave 2 | 12,535 | 11,914 | 622 | 0.050 | 0.310 | 0.068 | 0.323 |
|  | Post-wave 2 | 4,754 | 4,501 | 253 | 0.053 | 0.292 | 0.056 | 0.174 |
|  | Wave 3 | 13,586 | 12,788 | 798 | 0.059 | 0.412 | 0.049 | 0.307 |
| 35-59 | Wave 1 | 41,907 | 36,811 | 5,098 | 0.122 | 0.418 | 0.115 | 0.383 |
|  | Post-wave 1 | 7,110 | 6,208 | 902 | 0.127 | 0.256 | 0.093 | 0.256 |
|  | Wave 2 | 49,582 | 43,437 | 6,145 | 0.124 | 0.407 | 0.166 | 0.476 |
|  | Post-wave 2 | 10,196 | 8,650 | 1,547 | 0.152 | 0.383 | 0.122 | 0.322 |
|  | Wave 3 | 47,630 | 40,235 | 7,397 | 0.155 | 0.454 | 0.154 | 0.475 |
| 60-64 | Wave 1 | 8,357 | 6,971 | 1,386 | 0.166 | 0.458 | 0.252 | 0.519 |
|  | Post-wave 1 | 1,391 | 1,152 | 239 | 0.172 | 0.331 | 0.159 | 0.364 |
|  | Wave 2 | 12,350 | 10,679 | 1,671 | 0.135 | 0.433 | 0.319 | 0.580 |
|  | Post-wave 2 | 2,347 | 1,915 | 432 | 0.184 | 0.440 | 0.224 | 0.486 |
|  | Wave 3 | 10,551 | 8,851 | 1,700 | 0.161 | 0.467 | 0.273 | 0.577 |
| 65-69 | Wave 1 | 6,433 | 5,439 | 994 | 0.155 | 0.412 | 0.315 | 0.545 |
|  | Post-wave 1 | 1,122 | 927 | 195 | 0.174 | 0.292 | 0.228 | 0.451 |
|  | Wave 2 | 10,345 | 9,089 | 1,256 | 0.121 | 0.440 | 0.387 | 0.606 |
|  | Post-wave 2 | 2,092 | 1,724 | 368 | 0.176 | 0.435 | 0.282 | 0.527 |
|  | Wave 3 | 9,515 | 8,021 | 1,494 | 0.157 | 0.439 | 0.321 | 0.571 |
| 70-74 | Wave 1 | 4,926 | 4,208 | 718 | 0.146 | 0.397 | 0.342 | 0.539 |
|  | Post-wave 1 | 936 | 783 | 153 | 0.163 | 0.294 | 0.257 | 0.438 |
|  | Wave 2 | 8,482 | 7,606 | 876 | 0.103 | 0.374 | 0.423 | 0.636 |
|  | Post-wave 2 | 1,727 | 1,417 | 310 | 0.180 | 0.403 | 0.280 | 0.523 |
|  | Wave 3 | 8,347 | 7,177 | 1,173 | 0.141 | 0.448 | 0.366 | 0.621 |
| 75+ | Wave 1 | 9,152 | 7,916 | 1,236 | 0.135 | 0.325 | 0.365 | 0.562 |
|  | Post-wave 1 | 1,603 | 1,314 | 289 | 0.180 | 0.215 | 0.278 | 0.488 |
|  | Wave 2 | 12,303 | 11,277 | 1,026 | 0.083 | 0.284 | 0.461 | 0.616 |
|  | Post-wave 2 | 2,727 | 2,247 | 480 | 0.176 | 0.302 | 0.347 | 0.496 |
|  | Wave 3 | 14,797 | 13,169 | 1,629 | 0.110 | 0.325 | 0.436 | 0.581 |
| **Public Sector only** | | | | | | | | |
| 0-14 | Wave 1 | 1,142 | 1,077 | 65 | 0.057 | 0.277 | 0.049 | 0.231 |
|  | Post-wave 1 | 341 | 319 | 22 | 0.065 | 0.455 | 0.038 | 0.136 |
|  | Wave 2 | 1,417 | 1,352 | 65 | 0.046 | 0.215 | 0.049 | 0.169 |
|  | Post-wave 2 | 742 | 710 | 32 | 0.043 | 0.375 | 0.044 | 0.063 |
|  | Wave 3 | 1,948 | 1,863 | 85 | 0.044 | 0.329 | 0.051 | 0.129 |
| 15-34 | Wave 1 | 7,985 | 7,796 | 189 | 0.024 | 0.360 | 0.062 | 0.370 |
|  | Post-wave 1 | 1,920 | 1,841 | 79 | 0.041 | 0.342 | 0.076 | 0.304 |
|  | Wave 2 | 8,157 | 7,954 | 203 | 0.025 | 0.281 | 0.089 | 0.360 |
|  | Post-wave 2 | 3,426 | 3,305 | 121 | 0.035 | 0.372 | 0.070 | 0.198 |
|  | Wave 3 | 8,604 | 8,343 | 261 | 0.030 | 0.418 | 0.065 | 0.287 |
| 35-59 | Wave 1 | 19,030 | 18,279 | 751 | 0.039 | 0.386 | 0.185 | 0.505 |
|  | Post-wave 1 | 3,238 | 3,095 | 143 | 0.044 | 0.413 | 0.163 | 0.378 |
|  | Wave 2 | 23,589 | 22,819 | 770 | 0.033 | 0.325 | 0.233 | 0.534 |
|  | Post-wave 2 | 4,992 | 4,741 | 251 | 0.050 | 0.410 | 0.198 | 0.363 |
|  | Wave 3 | 20,097 | 19,241 | 856 | 0.043 | 0.405 | 0.242 | 0.541 |
| 60-64 | Wave 1 | 4,336 | 4,142 | 194 | 0.045 | 0.402 | 0.351 | 0.649 |
|  | Post-wave 1 | 653 | 621 | 32 | 0.049 | 0.469 | 0.246 | 0.531 |
|  | Wave 2 | 6,797 | 6,597 | 200 | 0.029 | 0.330 | 0.402 | 0.625 |
|  | Post-wave 2 | 1,239 | 1,165 | 74 | 0.060 | 0.446 | 0.311 | 0.635 |
|  | Wave 3 | 5,115 | 4,961 | 154 | 0.030 | 0.448 | 0.374 | 0.623 |
| 65-69 | Wave 1 | 3,660 | 3,534 | 126 | 0.034 | 0.310 | 0.409 | 0.690 |
|  | Post-wave 1 | 598 | 568 | 30 | 0.050 | 0.267 | 0.333 | 0.500 |
|  | Wave 2 | 6,159 | 6,054 | 105 | 0.017 | 0.400 | 0.458 | 0.571 |
|  | Post-wave 2 | 1,225 | 1,169 | 56 | 0.046 | 0.482 | 0.376 | 0.661 |
|  | Wave 3 | 4,826 | 4,707 | 119 | 0.025 | 0.538 | 0.428 | 0.571 |
| 70-74 | Wave 1 | 2,712 | 2,666 | 46 | 0.017 | 0.457 | 0.446 | 0.717 |
|  | Post-wave 1 | 482 | 456 | 26 | 0.054 | 0.308 | 0.366 | 0.577 |
|  | Wave 2 | 5,231 | 5,155 | 76 | 0.015 | 0.237 | 0.484 | 0.632 |
|  | Post-wave 2 | 947 | 901 | 46 | 0.049 | 0.457 | 0.363 | 0.565 |
|  | Wave 3 | 4,064 | 3,973 | 91 | 0.022 | 0.418 | 0.469 | 0.604 |
| 75+ | Wave 1 | 4,629 | 4,557 | 72 | 0.016 | 0.486 | 0.423 | 0.750 |
|  | Post-wave 1 | 768 | 744 | 24 | 0.031 | 0.375 | 0.378 | 0.583 |
|  | Wave 2 | 7,253 | 7,165 | 88 | 0.012 | 0.227 | 0.515 | 0.580 |
|  | Post-wave 2 | 1,314 | 1,274 | 40 | 0.030 | 0.425 | 0.446 | 0.500 |
|  | Wave 3 | 6,280 | 6,167 | 113 | 0.018 | 0.363 | 0.523 | 0.540 |

**Table C. Adjusted risk factors associated with mortality (non-ICU and ICU), treatment in ICU and mechanical ventilation, adjusting for race**

| **Characteristic** | **All sectors** | **Public sector** | **Private sector** | **All sectors** | **Public sector** | **Private sector** |
| --- | --- | --- | --- | --- | --- | --- |
|  | *ICU treatment* | | | *Mechanical ventilation* | | |
| Wave period (reference: between-wave period) | 0.91 (0.87-0.95) | 0.69 (0.62-0.76) | 0.97 (0.93-1.02) | 1.16 (1.09-1.23) | 1.00 (0.91-1.10) | 1.27 (1.17-1.38) |
| Public sector (reference: Private sector) | 0.17 (0.16-0.18) | **-** | - | 1.19 (1.14-1.24) | - | - |
| Male (reference: female) | 1.26 (1.23-1.30) | 1.16 (1.08-1.25) | 1.27 (1.24-1.31) | 1.00 (0.97-1.04) | 0.98 (0.91-1.04) | 1.00 (0.96-1.04) |
| Age groups (reference: 35-59 year olds) |  |  |  |  |  |  |
| 0-14 | 0.50 (0.43-0.57) | 1.24 (1.01-1.50) | 0.24 (0.19-0.31) | 0.89 (0.74-1.05) | 0.89 (0.74-1.06) | 0.66 (0.44-1.00) |
| 15-34 | 0.50 (0.47-0.53) | 0.72 (0.64-0.82) | 0.42 (0.38-0.45) | 0.90 (0.82-0.97) | 0.89 (0.79-1.00) | 0.87 (0.78-0.98) |
| 60-64 | 1.26 (1.21-1.32) | 0.98 (0.87-1.10) | 1.33 (1.27-1.38) | 1.04 (1.00-1.09) | 0.99 (0.90-1.09) | 1.06 (1.01-1.12) |
| 65-69 | 1.24 (1.18-1.30) | 0.72 (0.63-0.83) | 1.39 (1.32-1.45) | 1.00 (0.95-1.06) | 0.98 (0.87-1.11) | 1.01 (0.95-1.07) |
| 70-74 | 1.09 (1.03-1.15) | 0.62 (0.53-0.73) | 1.25 (1.18-1.32) | 0.98 (0.92-1.05) | 0.90 (0.77-1.06) | 1.00 (0.93-1.07) |
| 75+ | 0.94 (0.90-0.99) |  | 1.08 (1.03-1.13) | 0.83 (0.77-0.89) | 0.85 (0.72-1.00) | 0.83 (0.77-0.89) |
| Race (reference: Black) |  |  |  |  |  |  |
| Coloured | 1.08 (1.02-1.15) | 1.47 (1.26-1.72) | 1.07 (1.01-1.14) | 1.07 (1.00-1.13) | 1.10 (0.97-1.24) | 1.01 (0.94-1.09) |
| Indian | 1.18 (1.12-1.24) | 1.92 (1.59-2.31) | 1.12 (1.07-1.17) | 0.83 (0.78-0.89) | 1.20 (1.01-1.43) | 0.79 (0.73-0.85) |
| White | 0.98 (0.94-1.01) | 2.10 (1.78-2.46) | 0.91 (0.88-0.94) | 0.85 (0.81-0.89) | 1.02 (0.89-1.17) | 0.83 (0.79-0.87) |
| Other | 1.12 (0.77-1.55) | 2.09 (1.36-3.05) | 0.51 (0.25-1.03) | 1.35 (1.07-1.71) | 1.39 (1.12-1.73) | 0.79 (0.25-2.49) |
| Any comorbidity (reference: no) | 1.26 (1.22-1.29) | 1.49 (1.36-1.64) | 1.22 (1.19-1.26) | 1.18 (1.14-1.23) | 0.97 (0.89-1.06) | 1.22 (1.18-1.27) |
| Province (reference: Western Cape) |  |  |  |  |  |  |
| Eastern Cape | 0.64 (0.60-0.68) | 0.24 (0.21-0.28) | 0.80 (0.74-0.85) | 1.09 (1.00-1.19) | 2.28 (1.84-2.84) | 0.86 (0.78-0.95) |
| Free State | 0.70 (0.64-0.75) | 0.35 (0.29-0.41) | 0.85 (0.78-0.92) | 1.81 (1.67-1.96) | 3.31 (2.70-4.06) | 1.49 (1.36-1.64) |
| Gauteng | 0.94 (0.89-1.00) | 0.48 (0.42-0.55) | 1.07 (1.02-1.13) | 1.37 (1.28-1.48) | 2.67 (2.19-3.27) | 1.14 (1.06-1.24) |
| KwaZulu-Natal | 0.73 (0.69-0.77) | 0.51 (0.45-0.59) | 0.80 (0.75-0.85) | 1.18 (1.09-1.28) | 1.76 (1.42-2.18) | 1.04 (0.95-1.13) |
| Limpopo | 0.52 (0.46-0.58) | 0.41 (0.34-0.50) | 0.48 (0.40-0.56) | 1.51 (1.34-1.71) | 2.73 (2.18-3.42) | 1.20 (0.99-1.47) |
| Mpumalanga | 0.93 (0.86-1.01) | 0.49 (0.40-0.60) | 1.09 (1.00-1.18) | 1.04 (0.92-1.17) | 2.93 (2.34-3.66) | 0.72 (0.63-0.84) |
| Northern Cape | 0.83 (0.74-0.92) | 0.48 (0.37-0.61) | 0.94 (0.83-1.05) | 1.87 (1.70-2.04) | 3.42 (2.75-4.26) | 1.55 (1.39-1.73) |
| North West | 0.66 (0.62-0.71) | 0.63 (0.54-0.73) | 0.65 (0.60-0.70) | 1.26 (1.15-1.38) | 1.66 (1.31-2.09) | 1.19 (1.08-1.32) |
|  |  | *Non-ICU mortality* |  |  | *ICU mortality* |  |
| Wave period (reference: between-wave period) | 1.29 (1.25-1.33) | 1.24 (1.20-1.29) | 1.84 (1.67-2.05) | 1.20 (1.14-1.27) | 1.29 (1.15-1.44) | 1.17 (1.10-1.24) |
| Public sector (reference: Private sector) | 2.40 (2.33-2.47) | - | - | 1.09 (1.05-1.14) | - | - |
| Male (reference: female) | 1.12 (1.10-1.14) | 1.09 (1.07-1.12) | 1.33 (1.27-1.39) | 1.03 (1.00-1.06) | 1.01 (0.94-1.08) | 1.04 (1.00-1.07) |
| Age groups (reference: 35-59 year olds) |  |  |  |  |  |  |
| 0-14 | 0.21 (0.17-0.25) | 0.23 (0.19-0.28) | 0.04 (0.01-0.10) | 0.44 (0.32-0.60) | 0.45 (0.31-0.65) | 0.33 (0.17-0.63) |
| 15-34 | 0.37 (0.35-0.40) | 0.38 (0.36-0.41) | 0.25 (0.20-0.30) | 0.72 (0.65-0.79) | 0.66 (0.56-0.78) | 0.73 (0.64-0.83) |
| 60-64 | 1.67 (1.62-1.72) | 1.56 (1.51-1.61) | 2.11 (1.95-2.27) | 1.23 (1.18-1.28) | 1.19 (1.09-1.31) | 1.23 (1.18-1.29) |
| 65-69 | 1.94 (1.89-2.00) | 1.80 (1.75-1.86) | 2.61 (2.41-2.83) | 1.30 (1.24-1.36) | 1.16 (1.04-1.30) | 1.33 (1.27-1.40) |
| 70-74 | 2.10 (2.04-2.16) | 1.91 (1.85-1.97) | 3.39 (3.13-3.67) | 1.35 (1.29-1.42) | 1.19 (1.05-1.34) | 1.39 (1.32-1.47) |
| 75+ | 2.41 (2.35-2.46) | 2.10 (2.05-2.16) | 5.19 (4.87-5.53) | 1.42 (1.36-1.48) | 1.18 (1.05-1.33) | 1.48 (1.41-1.55) |
| Race (reference: black) |  |  |  |  |  |  |
| Coloured | 0.94 (0.90-0.97) | 0.92 (0.88-0.96) | 1.01 (0.91-1.12) | 1.07 (1.01-1.13) | 0.97 (0.84-1.12) | 1.11 (1.04-1.18) |
| Indian | 1.00 (0.95-1.05) | 0.99 (0.93-1.06) | 0.96 (0.89-1.05) | 1.03 (0.98-1.08) | 0.98 (0.84-1.13) | 1.04 (0.99-1.10) |
| White | 0.97 (0.94-1.00) | 0.91 (0.86-0.96) | 0.85 (0.80-0.90) | 1.01 (0.97-1.04) | 0.91 (0.77-1.07) | 1.01 (0.97-1.05) |
| Other | 0.85 (0.72-1.01) | 0.78 (0.65-0.94) | 1.64 (0.89-2.73) | 0.82 (0.52-1.30) | 0.84 (0.51-1.38) | 0.60 (0.19-1.87) |
| Any comorbidity (reference: no) | 1.31 (1.28-1.34) | 1.29 (1.26-1.33) | 1.30 (1.24-1.36) | 1.15 (1.11-1.18) | 1.13 (1.03-1.24) | 1.14 (1.11-1.18) |
| Province (reference: Western Cape) |  |  |  |  |  |  |
| Eastern Cape | 1.46 (1.39-1.53) | 1.47 (1.39-1.56) | 2.03 (1.83-2.27) | 1.47 (1.37-1.58) | 1.12 (0.95-1.31) | 1.60 (1.48-1.73) |
| Free State | 1.09 (1.03-1.15) | 1.10 (1.04-1.18) | 1.37 (1.20-1.57) | 1.46 (1.35-1.58) | 1.13 (0.96-1.33) | 1.62 (1.48-1.77) |
| Gauteng | 1.29 (1.23-1.36) | 1.39 (1.31-1.48) | 1.22 (1.11-1.36) | 1.13 (1.06-1.21) | 1.05 (0.91-1.21) | 1.20 (1.12-1.30) |
| KwaZulu-Natal | 1.32 (1.26-1.39) | 1.36 (1.28-1.44) | 1.47 (1.32-1.64) | 1.41 (1.32-1.51) | 1.28 (1.13-1.46) | 1.48 (1.37-1.60) |
| Limpopo | 1.45 (1.37-1.53) | 1.45 (1.36-1.55) | 3.01 (2.57-3.51) | 1.48 (1.32-1.65) | 1.12 (0.94-1.34) | 1.83 (1.59-2.12) |
| Mpumalanga | 1.50 (1.41-1.59) | 1.58 (1.48-1.69) | 1.39 (1.19-1.63) | 1.35 (1.24-1.47) | 1.08 (0.90-1.31) | 1.47 (1.33-1.62) |
| Northern Cape | 1.21 (1.13-1.31) | 1.25 (1.15-1.36) | 1.44 (1.18-1.75) | 1.04 (0.91-1.19) | 0.91 (0.70-1.18) | 1.11 (0.95-1.30) |
| North West | 0.98 (0.93-1.04) | 0.99 (0.92-1.06) | 1.12 (0.98-1.27) | 1.35 (1.24-1.46) | 1.08 (0.93-1.25) | 1.50 (1.36-1.64) |

**Table D. Adjusted coefficients for factors associated with mortality (non-ICU and ICU), treatment in ICU and mechanical ventilation, including an interaction term for wave period and sector, and province and sector**

| **Variable** | *ICU treatment* | *Mechanical ventilation* | *Non-ICU mortality* | *ICU mortality* |
| --- | --- | --- | --- | --- |
| Intercept | -1.54 (-1.58; -1.5) | -1.11 (-1.17; -1.06) | -3.5 (-3.57; -3.43) | -1.38 (-1.43; -1.33) |
| Wave period (reference: between-wave period) | -0.15 (-0.18; -0.12) | 0.22 (0.18; 0.27) | 0.74 (0.68; 0.81) | 0.31 (0.27; 0.34) |
| Public sector (reference: Private sector) | -1.31 (-1.4; -1.21) | -1.77 (-1.97; -1.59) | 1.27 (1.19; 1.35) | 0.28 (0.17; 0.39) |
| Interaction: Wave period x Sector |  |  |  |  |
| Wave period x Public | -0.29 (-0.38; -0.20) | -0.19 (-0.30; -0.07) | -0.51 (-0.58; -0.44) | -0.06 (-0.16; 0.05) |
| Male (reference: female) | 0.24 (0.22; 0.26) | -0.01 (-0.03; 0.02) | 0.16 (0.14; 0.17) | 0.04 (0.01; 0.06) |
| Age groups (reference: 35-59 year olds) |  |  |  |  |
| 0-14 | -0.93 (-1.03; -0.83) | -0.26 (-0.41; -0.12) | -1.76 (-1.92; -1.61) | -1.18 (-1.39; -0.98) |
| 15-34 | -0.7 (-0.74; -0.65) | -0.15 (-0.22; -0.09) | -0.99 (-1.03; -0.94) | -0.41 (-0.48; -0.35) |
| 60-64 | 0.22 (0.19; 0.25) | 0.04 (0.00; 0.07) | 0.56 (0.53; 0.58) | 0.22 (0.19; 0.25) |
| 65-69 | 0.22 (0.19; 0.25) | -0.02 (-0.06; 0.02) | 0.73 (0.71; 0.76) | 0.27 (0.24; 0.31) |
| 70-74 | 0.10 (0.07; 0.14) | -0.06 (-0.1; -0.01) | 0.86 (0.83; 0.89) | 0.32 (0.29; 0.36) |
| 75+ | -0.12 (-0.15; -0.09) | -0.32 (-0.37; -0.28) | 1.09 (1.07; 1.12) | 0.32 (0.29; 0.36) |
| Any comorbidity (reference: no) | 0.13 (0.11; 0.15) | 0.23 (0.20; 0.25) | 0.23 (0.21; 0.25) | 0.08 (0.06; 0.11) |
| Province (reference: Western Cape) |  |  |  |  |
| Eastern Cape | -0.12 (-0.17; -0.07) | -0.07 (-0.14; -0.01) | 0.51 (0.45; 0.57) | 0.40 (0.35; 0.45) |
| Free State | -0.21 (-0.26; -0.16) | 0.25 (0.19; 0.30) | 0.04 (-0.03; 0.11) | 0.34 (0.28; 0.39) |
| Gauteng | 0.19 (0.16; 0.22) | -0.08 (-0.12; -0.05) | 0.09 (0.05; 0.14) | 0.12 (0.08; 0.16) |
| KwaZulu-Natal | -0.14 (-0.17; -0.10) | -0.21 (-0.26; -0.16) | 0.25 (0.20; 0.30) | 0.29 (0.25; 0.33) |
| Limpopo | -0.51 (-0.58; -0.44) | 0.26 (0.18; 0.34) | 0.68 (0.62; 0.75) | 0.51 (0.44; 0.58) |
| Mpumalanga | -0.17 (-0.23; -0.12) | -0.05 (-0.13; 0.02) | 0.18 (0.10; 0.26) | 0.31 (0.25; 0.37) |
| Northern Cape | -0.31 (-0.39; -0.22) | 0.47 (0.39; 0.55) | 0.13 (0.03; 0.23) | 0.28 (0.19; 0.36) |
| North West | -0.27 (-0.33; -0.22) | 0.09 (0.02; 0.15) | -0.13 (-0.2; -0.05) | 0.26 (0.19; 0.31) |
| Interaction: Province x Sector |  |  |  |  |
| Eastern Cape x Public | -0.97 (-1.11; -0.84) | 2.21 (2.00; 2.42) | -0.21 (-0.27; -0.14) | -0.25 (-0.40; -0.11) |
| Free State x Public | -0.55 (-0.70; -0.41) | 2.21 (2.00; 2.41) | -0.02 (-0.10; 0.06) | -0.19 (-0.35; -0.04) |
| Gauteng x Public | -0.56 (-0.66; -0.46) | 2.36 (2.18; 2.55) | 0.18 (0.12; 0.23) | -0.06 (-0.17; 0.05) |
| KwaZulu-Natal x Public | -0.15 (-0.25; -0.06) | 2.10 (1.91; 2.29) | -0.01 (-0.07; 0.05) | -0.02 (-0.13; 0.08) |
| Limpopo x Public | -0.10 (-0.27; 0.07) | 2.01 (1.78; 2.24) | -0.37 (-0.45; -0.29) | -0.39 (-0.57; -0.21) |
| Mpumalanga x Public | -0.25 (-0.43; -0.08) | 2.37 (2.14; 2.61) | 0.24 (0.15; 0.34) | -0.23 (-0.42; -0.05) |
| Northern Cape x Public | 0.06 (-0.18; 0.28) | 2.05 (1.80; 2.31) | 0.00 (-0.12; 0.13) | -0.36 (-0.62; -0.12) |
| North West x Public | 0.17 (0.05; 0.30) | 1.70 (1.49; 1.92) | 0.05 (-0.04; 0.14) | -0.22 (-0.35; -0.08) |

**Table E. Adjusted risk factors associated with mortality (non-ICU and ICU), treatment in ICU and mechanical ventilation including individual comorbidities**

| **Characteristic** | **All sectors** | **Public sector** | **Private sector** | **All sectors** | **Public sector** | **Private sector** |
| --- | --- | --- | --- | --- | --- | --- |
|  | *ICU treatment* | | | *Mechanical ventilation* | | |
| Wave period (reference: between-wave period) | 0.84 (0.82-0.87) | 0.70 (0.62-0.80) | 0.85 (0.83-0.88) | 1.25 (1.20-1.31) | 0.95 (0.78-1.17) | 1.27 (1.21-1.33) |
| Public sector (reference: Private sector) | 0.15 (0.14-0.15) | **-** | **-** | 0.73 (0.68-0.78) | **-** | **-** |
| Male (reference: female) | 1.27 (1.25-1.30) | 1.15 (1.05-1.26) | 1.28 (1.25-1.31) | 0.99 (0.96-1.02) | 1.01 (0.88-1.17) | 0.99 (0.96-1.01) |
| Age groups (reference: 35-59 year olds) |  |  |  |  |  |  |
| 0-14 | 0.35 (0.31-0.39) | 1.54 (1.19-1.95) | 0.28 (0.24-0.31) | 0.89 (0.75-1.04) | 1.22 (0.90-1.64) | 0.65 (0.52-0.80) |
| 15-34 | 0.47 (0.45-0.50) | 0.78 (0.67-0.90) | 0.44 (0.42-0.46) | 0.85 (0.79-0.91) | 0.83 (0.66-1.03) | 0.83 (0.77-0.90) |
| 60-64 | 1.25 (1.21-1.29) | 0.87 (0.75-1.00) | 1.27 (1.23-1.31) | 1.05 (1.01-1.09) | 1.02 (0.82-1.26) | 1.05 (1.01-1.09) |
| 65-69 | 1.26 (1.22-1.03) | 0.47 (0.39-0.56) | 1.33 (1.28-1.37) | 1.00 (0.96-1.05) | 0.94 (0.69-1.26) | 1.00 (0.96-1.05) |
| 70-74 | 1.13 (1.09-1.17) | 0.39 (0.31-0.49) | 1.19 (1.15-1.24) | 0.98 (0.93-1.03) | 0.92 (0.64-1.27) | 0.97 (0.92-1.02) |
| 75+ | 0.90 (0.87-0.93) | 0.23 (0.17-0.29) | 0.95 (0.92-0.98) | 0.76 (0.72-0.79) | 0.83 (0.56-1.19) | 0.74 (0.71-0.78) |
| Hypertension (reference: no) | 1.04 (1.01-1.06) | 1.01 (0.91-1.12) | 1.05 (1.03-1.08) | 1.13 (1.10-1.17) | 1.14 (0.98-1.33) | 1.11 (1.07-1.14) |
| Diabetes (reference: no) | 1.24 (1.20-1.27) | 1.63 (1.47-1.82) | 1.19 (1.16-1.22) | 1.14 (1.11-1.19) | 1.10 (0.94-1.29) | 1.19 (1.15-1.24) |
| Cardiac Disease (reference: no) | 1.09 (1.01-1.18) | 1.50 (1.14-1.95) | 1.07 (0.99-1.15) | 1.01 (0.92-1.12) | 0.71 (0.49-1.01) | 0.98 (0.88-1.09) |
| Chronic Pulmonary Disease (reference: no) | 1.01 (0.92-1.10) | 1.33 (0.99-1.77) | 1.00 (0.90-1.11) | 0.83 (0.72-0.95) | 0.54 (0.33-0.83) | 1.10 (0.95-1.27) |
| Asthma (reference: no) | 1.03 (0.97-1.09) | 0.63 (0.47-0.85) | 1.06 (1.00-1.13) | 1.01 (0.93-1.09) | 1.02 (0.66-1.51) | 1.04 (0.96-1.12) |
| Chronic Renal Failure (reference: no) | 1.08 (0.97-1.19) | 0.89 (0.72-1.09) | 1.28 (1.14-1.42) | 1.10 (0.96-1.24) | 0.98 (0.68-1.36) | 1.16 (1.01-1.33) |
| Malignancy (reference: no) | 1.01 (0.87-1.16) | 1.52 (0.89-2.40) | 0.97 (0.84-1.12) | 0.77 (0.61-0.96) | 0.69 (0.30-1.33) | 0.74 (0.58-0.93) |
| Tuberculosis (reference: no) | 0.89 (0.75-1.04) | 0.63 (0.42-0.90) | 0.97 (0.82-1.15) | 1.13 (0.91-1.38) | 1.18 (0.61-2.07) | 1.06 (0.85-1.32) |
| Tuberculosis in past (reference: no) | 0.78 (0.65-0.92) | 0.68 (0.55-0.83) | 0.37 (0.18-0.67) | 0.61 (0.44-0.83) | 1.07 (0.70-1.57) | 1.54 (0.71-2.88) |
| HIV positive (reference: no) | 0.99 (0.92-1.07) | 0.96 (0.84-1.10) | 0.90 (0.81-0.98) | 1.28 (1.16-1.41) | 1.00 (0.81-1.24) | 1.42 (1.27-1.59) |
| Provinces (reference: Western Cape) |  |  |  |  |  |  |
| Eastern Cape | 0.79 (0.75-0.83) | 0.32 (0.27-0.38) | 1.05 (1.03-1.08) | 1.10 (1.04-1.17) | 7.57 (5.81-9.89) | 0.93 (0.87-0.99) |
| Free State | 0.71 (0.68-0.75) | 0.30 (0.24-0.37) | 1.19 (1.16-1.22) | 1.43 (1.34-1.52) | 9.24 (6.99-12.19) | 1.22 (1.14-1.30) |
| Gauteng | 1.16 (1.13-1.20) | 0.69 (0.58-0.83) | 1.07 (0.99-1.15) | 1.01 (0.97-1.05) | 7.85 (6.04-10.22) | 0.89 (0.86-0.93) |
| KwaZulu-Natal | 0.85 (0.82-0.88) | 0.66 (0.57-0.77) | 1.00 (0.90-1.11) | 0.92 (0.87-0.96) | 5.93 (4.64-7.61) | 0.79 (0.75-0.83) |
| Limpopo | 0.60 (0.56-0.65) | 0.61 (0.46-0.79) | 1.06 (1.00-1.13) | 1.48 (1.36-1.60) | 7.30 (5.11-10.28) | 1.29 (1.18-1.40) |
| Mpumalanga | 0.79 (0.74-0.83) | 0.47 (0.30-0.70) | 1.28 (1.14-1.42) | 1.04 (0.96-1.12) | 8.88 (5.40-13.96) | 0.90 (0.83-0.98) |
| Northern Cape | 0.70 (0.64-0.76) | 0.99 (0.67-1.40) | 0.97 (0.84-1.12) | 1.87 (1.71-2.03) | 11.27 (7.64-16.26) | 1.61 (1.47-1.75) |
| North West | 0.78 (0.73-0.82) | 0.50 (0.37-0.67) | 0.97 (0.82-1.15) | 1.27 (1.18-1.36) | 9.89 (7.00-13.78) | 1.09 (1.02-1.17) |
|  |  | *Non-ICU mortality* |  |  | *ICU mortality* |  |
| Wave period (reference: between-wave period) | 1.59 (1.52-1.65) | 1.28 (1.22-1.35) | 2.20 (2.04-2.37) | 1.38 (1.33-1.44) | 1.19 (1.04-1.37) | 1.40 (1.34-1.45) |
| Public sector (reference: Private sector) | 2.08 (2.03-2.13) | - | - | 1.21 (1.15-1.28) | - | - |
| Male (reference: female) | 1.19 (1.17-1.22) | 1.15 (1.12-1.18) | 1.25 (1.21-1.29) | 1.03 (1.01-1.06) | 1.00 (0.92-1.10) | 1.04 (1.01-1.06) |
| Age groups (reference: 35-59 year olds) |  |  |  |  |  |  |
| 0-14 | 0.16 (0.13-0.19) | 0.24 (0.19-0.29) | 0.04 (0.02-0.07) | 0.27 (0.21-0.34) | 0.39 (0.26-0.56) | 0.22 (0.15-0.30) |
| 15-34 | 0.33 (0.31-0.36) | 0.34 (0.31-0.36) | 0.28 (0.25-0.32) | 0.66 (0.62-0.71) | 0.68 (0.58-0.80) | 0.66 (0.61-0.71) |
| 60-64 | 1.89 (1.83-1.95) | 1.65 (1.58-1.72) | 2.11 (2.00-2.24) | 1.26 (1.22-1.30) | 1.25 (1.11-1.41) | 1.26 (1.22-1.30) |
| 65-69 | 2.26 (2.18-2.33) | 1.88 (1.80-1.96) | 2.70 (2.55-2.85) | 1.32 (1.28-1.37) | 1.29 (1.09-1.51) | 1.33 (1.28-1.38) |
| 70-74 | 2.71 (2.62-2.80) | 2.12 (2.03-2.21) | 3.56 (3.37-3.76) | 1.40 (1.35-1.46) | 1.22 (1.00-1.48) | 1.41 (1.36-1.47) |
| 75+ | 3.58 (3.49-3.68) | 2.33 (2.25-2.43) | 5.46 (5.24-5.69) | 1.41 (1.36-1.46) | 1.17 (0.92-1.47) | 1.42 (1.37-1.47) |
| Hypertension (reference: no) | 1.01 (0.99-1.03) | 1.07 (1.04-1.10) | 0.97 (0.93-1.01) | 1.03 (1.00-1.06) | 1.01 (0.92-1.11) | 1.04 (1.01-1.07) |
| Diabetes (reference: no) | 1.25 (1.22-1.28) | 1.23 (1.19-1.26) | 1.24 (1.19-1.30) | 1.11 (1.08-1.15) | 1.15 (1.04-1.27) | 1.10 (1.07-1.14) |
| Cardiac Disease (reference: no) | 1.06 (1.00-1.12) | 1.07 (1.00-1.14) | 1.06 (0.95-1.18) | 0.94 (0.86-1.01) | 0.86 (0.65-1.11) | 0.96 (0.88-1.04) |
| Chronic Pulmonary Disease (reference: no) | 1.03 (0.97-1.09) | 0.86 (0.80-0.93) | 1.51 (1.34-1.69) | 1.02 (0.92-1.12) | 0.67 (0.51-0.87) | 1.08 (0.96-1.21) |
| Asthma (reference: no) | 0.84 (0.79-0.89) | 0.96 (0.90-1.03) | 0.82 (0.73-0.91) | 0.99 (0.93-1.06) | 1.34 (1.03-1.74) | 0.99 (0.92-1.05) |
| Chronic Renal Failure (reference: no) | 1.20 (1.15-1.26) | 1.27 (1.22-1.33) | 1.49 (1.26-1.74) | 1.18 (1.06-1.29) | 1.19 (0.99-1.41) | 1.15 (1.02-1.29) |
| Malignancy (reference: no) | 1.29 (1.16-1.43) | 1.09 (0.95-1.25) | 1.60 (1.35-1.87) | 1.10 (0.95-1.27) | 1.16 (0.75-1.72) | 1.11 (0.94-1.29) |
| Tuberculosis (reference: no) | 1.21 (1.10-1.31) | 1.17 (1.06-1.27) | 1.39 (1.07-1.77) | 1.07 (0.89-1.27) | 0.87 (0.55-1.32) | 1.12 (0.92-1.35) |
| Tuberculosis in past (reference: no) | 1.11 (1.05-1.18) | 1.09 (1.03-1.15) | 0.72 (0.33-1.35) | 0.87 (0.70-1.07) | 0.81 (0.65-1.01) | 1.21 (0.55-2.29) |
| HIV positive (reference: no) | 1.26 (1.21-1.31) | 1.11 (1.07-1.16) | 1.48 (1.28-1.70) | 1.13 (1.04-1.23) | 1.15 (1.02-1.31) | 1.10 (0.99-1.22) |
| Provinces (reference: Western Cape) |  |  |  |  |  |  |
| Eastern Cape | 1.36 (1.32-1.40) | 1.28 (1.23-1.32) | 1.75 (1.64-1.87) | 1.41 (1.34-1.48) | 1.04 (0.87-1.23) | 1.47 (1.39-1.55) |
| Free State | 0.99 (0.96-1.03) | 0.98 (0.93-1.02) | 1.10 (1.02-1.19) | 1.30 (1.23-1.37) | 1.05 (0.86-1.28) | 1.35 (1.27-1.43) |
| Gauteng | 1.01 (0.98-1.05) | 1.02 (0.96-1.08) | 1.11 (1.06-1.17) | 1.07 (1.04-1.11) | 0.96 (0.80-1.14) | 1.10 (1.06-1.15) |
| KwaZulu-Natal | 1.17 (1.13-1.21) | 1.12 (1.07-1.17) | 1.38 (1.30-1.46) | 1.27 (1.22-1.32) | 1.12 (0.98-1.28) | 1.31 (1.25-1.36) |
| Limpopo | 1.51 (1.43-1.59) | 1.23 (1.14-1.31) | 2.10 (1.94-2.27) | 1.56 (1.45-1.67) | 1.03 (0.79-1.32) | 1.65 (1.53-1.77) |
| Mpumalanga | 1.19 (1.12-1.27) | 1.40 (1.27-1.53) | 1.28 (1.17-1.40) | 1.30 (1.22-1.38) | 0.57 (0.31-0.95) | 1.35 (1.27-1.44) |
| Northern Cape | 1.01 (0.93-1.10) | 0.86 (0.74-0.99) | 1.21 (1.08-1.35) | 1.23 (1.12-1.34) | 0.70 (0.46-1.02) | 1.30 (1.19-1.43) |
| North West | 0.96 (0.91-1.02) | 1.10 (1.00-1.20) | 0.97 (0.89-1.06) | 1.25 (1.18-1.33) | 0.97 (0.72-1.26) | 1.30 (1.22-1.38) |

**Table F. Adjusted risk factors associated with mortality (non-ICU and ICU), treatment in ICU and mechanical ventilation, stratified by age group**

|  | **0-14 years** | **15-34 years** | **35-59 years** | | **60-64 years** | **65-69 years** | **70-74 years** | **75+ years** |
| --- | --- | --- | --- | --- | --- | --- | --- | --- |
| *ICU treatment* |  |  |  | |  |  |  |  |
| Wave period (reference: between-wave period) | 1.26 (0.95-1.68) | 0.91 (0.81-1.02) | 0.90 (0.87-0.94) | | 0.90 (0.84-0.96) | 0.82 (0.76-0.89) | 0.77 (0.70-0.83) | 0.62 (0.58-0.66) |
| Public sector (reference: Private sector) | 0.66 (0.53-0.83) | 0.25 (0.22-0.28) | 0.19 (0.18-0.19) | | 0.14 (0.13-0.15) | 0.10 (0.09-0.11) | 0.08 (0.07-0.10) | 0.08 (0.07-0.10) |
| Male (reference: female) | 0.96 (0.78-1.17) | 1.72 (1.58-1.87) | 1.28 (1.25-1.32) | | 1.25 (1.19-1.31) | 1.15 (1.10-1.22) | 1.28 (1.20-1.36) | 1.29 (1.22-1.36) |
| Any comorbidity (reference: no) | 1.82 (1.40-2.36) | 1.65 (1.50-1.82) | 1.16 (1.13-1.19) | | 1.04 (1.00-1.10) | 0.99 (0.94-1.04) | 0.98 (0.92-1.04) | 0.94 (0.89-0.99) |
| *Mechanical ventilation* |  |  |  | |  |  |  |  |
| Wave period (reference: between-wave period) | 0.85 (0.58-1.25) | 1.55 (1.26-1.91) | 1.27 (1.19-1.35) | | 1.14 (1.03-1.26) | 1.13 (1.00-1.27) | 1.13 (0.99-1.29) | 1.19 (1.04-1.35) |
| Public sector (reference: Private sector) | 2.04 (1.47-2.84) | 0.97 (0.82-1.14) | 0.77 (0.73-0.82) | | 0.75 (0.67-0.85) | 0.83 (0.72-0.95) | 0.90 (0.75-1.08) | 1.16 (0.97-1.39) |
| Male (reference: female) | 1.10 (0.81-1.49) | 0.98 (0.86-1.11) | 1.00 (0.96-1.03) | | 0.99 (0.93-1.06) | 0.96 (0.89-1.03) | 0.99 (0.91-1.08) | 1.01 (0.92-1.10) |
| Any comorbidity (reference: no) | 0.72 (0.49-1.08) | 1.17 (1.01-1.36) | 1.31 (1.27-1.36) | | 1.28 (1.20-1.36) | 1.30 (1.21-1.40) | 1.17 (1.08-1.28) | 1.22 (1.11-1.33) |
| *Non-ICU mortality* | | | |  | | | | |
| Wave period (reference: between-wave period) | 1.36 (0.83-2.22) | 1.15 (1.00-1.31) | 1.44 (1.36-1.53) | | 1.47 (1.34-1.60) | 1.34 (1.24-1.45) | 1.38 (1.28-1.50) | 1.39 (1.31-1.47) |
| Public sector (reference: Private sector) | 15.74 (7.63-32.47) | 3.15 (2.73-3.63) | 3.00 (2.89-3.12) | | 2.50 (2.36-2.64) | 2.42 (2.29-2.55) | 2.01 (1.91-2.11) | 1.54 (1.49-1.59) |
| Male (reference: female) | 0.80 (0.56-1.13) | 1.42 (1.28-1.56) | 1.17 (1.13-1.20) | | 1.16 (1.11-1.21) | 1.10 (1.06-1.15) | 1.11 (1.07-1.15) | 1.14 (1.11-1.17) |
| Any comorbidity (reference: no) | 1.98 (1.38-2.84) | 2.98 (2.68-3.32) | 1.45 (1.40-1.51) | | 1.16 (1.10-1.22) | 1.03 (0.98-1.08) | 1.03 (0.98-1.08) | 1.04 (1.01-1.07) |
| *ICU mortality* | | | |  | | | | |
| Wave period (reference: between-wave period) | 2.58 (0.83-7.99) | 1.73 (1.35-2.22) | 1.53 (1.43-1.63) | | 1.28 (1.17-1.41) | 1.16 (1.06-1.27) | 1.22 (1.10-1.35) | 1.18 (1.09-1.27) |
| Public sector (reference: Private sector) | 2.03 (1.12-3.66) | 1.14 (0.96-1.36) | 1.14 (1.08-1.19) | | 1.12 (1.04-1.21) | 1.06 (0.96-1.16) | 1.06 (0.94-1.18) | 1.08 (0.97-1.20) |
| Male (reference: female) | 1.15 (0.68-1.94) | 0.93 (0.81-1.08) | 1.01 (0.98-1.04) | | 1.06 (1.01-1.12) | 0.98 (0.93-1.04) | 1.03 (0.97-1.09) | 1.10 (1.05-1.16) |
| Any comorbidity (reference: no) | 1.33 (0.73-2.43) | 1.21 (1.03-1.43) | 1.13 (1.09-1.16) | | 1.07 (1.01-1.12) | 1.08 (1.02-1.14) | 1.01 (0.95-1.07) | 1.12 (1.06-1.18) |

**Table G.** **Median and interquartile ranges for days in hospital in the intensive care unit (ICU) and non-ICU wards to death and recovery, stratified by age and time period across all provinces and sectors**

|  |  | **Median days (interquartile range) [n]** | | | | | |
| --- | --- | --- | --- | --- | --- | --- | --- |
| **Age (years)** | **Time period** | **Non-ICU to death** | **Non-ICU to recovery** | **Non-ICU to ICU** | **ICU to death** | **ICU to recovery** | **Post-ICU to recovery** |
| 0-14 | Wave 1 | 4 (1-11) [n=56] | 5 (2-9) [n=1,689] | 0 (0-0) [n=113] | 10.5 (4.75-29.25) [n=22] | 9 (4-19) [n=87] | 0 (0-2) [n=87] |
|  | Post-wave 1 | 1 (1-5.75) [n=12] | 3 (2-8) [n=652] | 0 (0-0) [n=36] | 6 (4-8) [n=2] | 10 (7-17) [n=29] | 0 (0-0) [n=29] |
|  | Wave 2 | 4 (1-8) [n=67] | 5 (2-9) [n=2,113] | 0 (0-0) [n=113] | 8 (3-19) [n=19] | 9.5 (4-22.75) [n=82] | 0 (0-0) [n=82] |
|  | Post-wave 2 | 2 (1-6.5) [n=31] | 5 (2-9) [n=1,017] | 0 (0-0) [n=47] | 3.5 (3.25-3.75) [n=2] | 6 (3-13) [n=37] | 0 (0-0) [n=37] |
|  | Wave 3 | 4 (1-10) [n=101] | 4 (2-7) [n=2,936] | 0 (0-2) [n=170] | 6 (2-19) [n=17] | 6 (3-11) [n=125] | 0 (0-0) [n=125] |
| 15-34 | Wave 1 | 4 (1-11) [n=528] | 6 (3-11) [n=11,727] | 0 (0-0) [n=177] | 14 (5-27.5) [n=140] | 7 (3-13) [n=334] | 0 (0-3) [n=334] |
|  | Post-wave 1 | 4 (1-10) [n=148] | 6 (3-10) [n=2,789] | 0 (0-0) [n=177] | 8.5 (4.8-18) [n=36] | 7 (4-14) [n=131] | 0 (0-1) [n=131] |
|  | Wave 2 | 4 (2-9) [n=815] | 6 (3-9) [n=10,826] | 0 (0-4) [n=566] | 8 (2-15) [n=190] | 9 (4-16) [n=357] | 0 (0-3) [n=357] |
|  | Post-wave 2 | 5.5 (2-12) [n=250] | 6 (3-10) [n=4,113] | 0 (0-2) [n=233] | 7 (2-11) [n=41] | 6 (3-10) [n=182] | 0 (0-2) [n=182] |
|  | Wave 3 | 4 (2-9) [n=629] | 5 (3-9) [n=11,487] | 0 (0-5) [n=737] | 9 (3-14) [n=227] | 7 (3-13) [n=443] | 0 (0-3) [n=443] |
| 35-59 | Wave 1 | 5 (1-12) [n=4,223] | 7 (4-12) [n=31,952] | 0 (0-4) [n=4,596] | 15 (6-32) [n=1,811] | 9 (5-17) [n=2,583] | 1 (0-6) [n=2,583] |
|  | Post-wave 1 | 5 (2-12.5) [n=583] | 7 (4-11) [n=5,446] | 0 (0-4) [n=843] | 11 (5-24) [n=215] | 8 (4-14) [n=588] | 0 (0-3) [n=588] |
|  | Wave 2 | 5 (2-9) [n=7,238] | 7 (4-11) [n=35,399] | 0 (0-6) [n=5,552] | 9 (5-17) [n=2,693] | 9 (5-17) [n=2,654] | 0 (0-5) [n=2,654] |
|  | Post-wave 2 | 5 (2-11) [n=1,058] | 7 (4-11) [n=7,309] | 0 (0-5) [n=1,392] | 10 (6-19) [n=437] | 8 (4-15) [n=872] | 0 (0-4) [n=872] |
|  | Wave 3 | 5 (2-9) [n=6,200] | 7 (4-11) [n=32,259] | 3 (0-6) [n=6,734] | 9 (5-16) [n=3,259] | 9 (5-16) [n=2,928] | 1 (0-5) [n=2,928] |
| 60-64 | Wave 1 | 4 (1-11) [n=1,758] | 8 (5-13) [n=5,076] | 0 (0-5) [n=1,229] | 14 (6.25-31) [n=662] | 8 (4-16) [n=511] | 2 (0-7) [n=511] |
|  | Post-wave 1 | 5 (2-11) [n=183] | 8 (5-12) [n=928] | 0 (0-5) [n=211] | 11 (4-22) [n=77] | 9 (4-16) [n=130] | 0 (0-3) [n=130] |
|  | Wave 2 | 4 (2-8.5) [n=3,423] | 8 (5-12) [n=7,065] | 0 (0-6) [n=1,496] | 10 (5-17) [n=892] | 11 (5-19) [n=536] | 0.5 (0-5) [n=536] |
|  | Post-wave 2 | 6 (2-12) [n=430] | 8 (5-12) [n=1,399] | 0 (0-5) [n=386] | 10 (5-17) [n=186] | 9 (5-19) [n=172] | 0 (0-5) [n=172] |
|  | Wave 3 | 5 (2-9) [n=2,419] | 8 (5-12) [n=6,027] | 3 (0-7) [n=1,530] | 10 (5-17) [n=893] | 9 (5-18) [n=518] | 1 (0-6) [n=518] |
| 65-69 | Wave 1 | 4 (1-11) [n=1,721] | 9 (5-14) [n=3,587] | 0 (0-5) [n=877] | 15 (7-34) [n=486] | 9 (4-18) [n=341] | 2 (0-7) [n=341] |
|  | Post-wave 1 | 4 (2-12.5) [n=211] | 8 (5-13) [n=688] | 0 (0-5) [n=172] | 11 (5-23) [n=79] | 9 (4-16) [n=86] | 0 (0-3) [n=86] |
|  | Wave 2 | 4 (1-9) [n=3,531] | 8 (5-13) [n=5,391] | 2 (0-7) [n=1,120] | 9 (5-15) [n=693] | 9 (4.8-16.3) [n=384] | 1 (0-6.3) [n=384] |
|  | Post-wave 2 | 5 (2-10) [n=488] | 9 (5-13) [n=1,173] | 0 (0-6) [n=333] | 12 (6-20.8) [n=178] | 7 (3-15.5) [n=135] | 1 (0-8) [n=135] |
|  | Wave 3 | 5 (2-9) [n=2,578] | 8 (5-13) [n=5,137] | 3 (0-7) [n=1,354] | 9 (4.25-17) [n=786] | 8 (4-16) [n=470] | 2 (0-7) [n=470] |
| 70-74 | Wave 1 | 4 (1-11) [n=1,444] | 9 (5-14) [n=2,697] | 0 (0-6) [n=642] | 12 (5-29) [n=348] | 7 (3-18) [n=264] | 2 (0-7) [n=264] |
|  | Post-wave 1 | 4 (2-9) [n=201] | 8 (5-13) [n=550] | 0 (0-5) [n=143] | 15 (5-22) [n=61] | 6.5 (4-15.3) [n=72] | 0 (0-6) [n=72] |
|  | Wave 2 | 4 (2-9) [n=3,234] | 9 (5-13) [n=4,227] | 0 (0-7) [n=797] | 8 (4-15) [n=504] | 8 (4-15) [n=241] | 1 (0-6) [n=241] |
|  | Post-wave 2 | 5 (2-10) [n=398] | 9 (5-13) [n=970] | 0 (0-5) [n=272] | 11 (6-21) [n=145] | 10 (6-16.8) [n=106] | 0 (0-5.8) [n=106] |
|  | Wave 3 | 5 (2-9) [n=2,633] | 9 (5-13) [n=4,279] | 3 (0-7) [n=1,057] | 9 (4-16) [n=655] | 9 (4-17) [n=301] | 1 (0-6) [n=301] |
| 75+ | Wave 1 | 6 (2-13) [n=2,896] | 9 (5-15) [n=4,890] | 0 (0-6) [n=1,101] | 10 (3-27.5) [n=631] | 8 (4-16) [n=366] | 1 (0-8) [n=366] |
|  | Post-wave 1 | 6 (2-11) [n=368] | 9 (5-14) [n=912] | 0 (0-5) [n=267] | 9 (4-19) [n=132] | 8.5 (4.8-15) [n=120] | 0 (0-5) [n=120] |
|  | Wave 2 | 5 (2-9) [n=5,219] | 9 (5-14) [n=5,846] | 0 (0-7) [n=926] | 7 (3-14) [n=585] | 7 (3-15) [n=258] | 0 (0-6) [n=258] |
|  | Post-wave 2 | 6 (2-11) [n=781] | 9 (5-14) [n=1,388] | 0 (0-5) [n=438] | 9 (4-18) [n=216] | 8 (3-13) [n=171] | 0 (0-5.5) [n=171] |
|  | Wave 3 | 6 (3-10) [n=5,743] | 9 (5-14) [n=6,959] | 2 (0-7) [n=1,496] | 7 (3-13) [n=875] | 7 (3-14) [n=414] | 1 (0-8) [n=414] |
| All ages | Wave 1 | 5 (2-12) [n=12,626] | 7 (4-12) [n=61,618] | 0 (0-5) [n=9,064] | 14 (5-31) [n=4,100] | 9 (4-17) [n=4,486] | 1 (0-6) [n=4,486] |
|  | Post-wave 1 | 5 (2-11) [n=1,706] | 7 (4-11) [n=11,965] | 0 (0-4) [n=1,849] | 10 (5-21) [n=602] | 8 (4-15) [n=1,156] | 0 (0-3) [n=1,156] |
|  | Wave 2 | 4 (2-9) [n=23,527] | 7 (4-11) [n=70,867] | 0 (0-6) [n=10,561] | 9 (4-16) [n=5,576] | 9 (5-17) [n=4,512] | 0 (0-5) [n=4,512] |
|  | Post-wave 2 | 5 (2-11) [n=3,436] | 7 (4-11) [n=17,369] | 0 (0-5) [n=3,101] | 10 (5-19) [n=1,205] | 8 (4-15) [n=1,675] | 0 (0-4) [n=1,675] |
|  | Wave 3 | 5 (2-9) [n=20,303] | 7 (4-11) [n=69,084] | 2 (0-7) [n=13,078] | 9 (4-16) [n=6,712] | 8 (4-16) [n=5,199] | 1 (0-5) [n=5,199] |

**Table H.** **Median and interquartile ranges for days in hospital in the intensive care unit (ICU) and non-ICU wards to death and recovery, stratified by public/private sector and time period across all age groups**

|  |  | **Median days (interquartile range) [n]** | | | | | |
| --- | --- | --- | --- | --- | --- | --- | --- |
| **Sector** | **Time period** | **Non-ICU to death** | **Non-ICU to recovery** | **Non-ICU to ICU** | **ICU to death** | **ICU to recovery** | **Post-ICU to recovery** |
| Public Sector | Wave 1 | 4 (1-9) [n=9,964] | 8 (4-13) [n=30,926] | 0 (0-0) [n=1,337] | 8 (3-16) [n=726] | 11 (6-20.25) [n=540] | 0 (0-0) [n=540] |
|  | Post-wave 1 | 4 (1-10) [n=1,449] | 8 (5-12) [n=5,823] | 0 (0-0) [n=333] | 7 (3-17.25) [n=136] | 12 (7-19) [n=157] | 0 (0-0) [n=157] |
|  | Wave 2 | 4 (1-8) [n=17,763] | 8 (5-12) [n=37,686] | 0 (0-2) [n=1,385] | 6 (3-11) [n=752] | 11 (6-19) [n=545] | 0 (0-0) [n=545] |
|  | Post-wave 2 | 5 (2-10) [n=2,902] | 8 (5-12) [n=9,800] | 0 (0-0) [n=573] | 6 (3-11) [n=232] | 8 (3-13) [n=258] | 0 (0-0) [n=258] |
|  | Wave 3 | 4 (2-8) [n=14,265] | 8 (4-12) [n=32,127] | 0 (0-2) [n=1,571] | 6 (3-11) [n=785] | 9 (4-15) [n=596] | 0 (0-0) [n=596] |
| Private Sector | Wave 1 | 14 (6-32) [n=2,662] | 7 (4-12) [n=30,692] | 0 (0-5) [n=7,718] | 16 (6-35) [n=3,371] | 8 (4-16) [n=3,941] | 2 (0-6) [n=3,941] |
|  | Post-wave 1 | 9 (4-20) [n=257] | 5 (3-9) [n=6,142] | 0 (0-5) [n=1,515] | 11 (5-23) [n=466] | 7 (4-14) [n=997] | 0 (0-4) [n=997] |
|  | Wave 2 | 6.5 (3-12) [n=5,764] | 7 (4-11) [n=33,181] | 2 (0-6) [n=9,181] | 9 (5-17) [n=4,825] | 9 (4-17) [n=3,966] | 1 (0-5) [n=3,966] |
|  | Post-wave 2 | 10 (5-16) [n=534] | 6 (3-10) [n=7,569] | 0 (0-5) [n=2,529] | 12 (6-21) [n=973] | 8 (4-15) [n=1,417] | 0 (0-5) [n=1,417] |
|  | Wave 3 | 7 (4-12) [n=6,038] | 7 (4-10) [n=36,957] | 3 (0-7) [n=11,494] | 9 (5-17) [n=5,922] | 8 (4-16) [n=4,594] | 2 (0-6) [n=3,941] |

**Table I.** **Median and interquartile ranges for days in hospital in the intensive care unit (ICU) and non-ICU wards to death and recovery, stratified by province and time period across all age groups**

|  |  | **Median days (interquartile range) [n]** | | | | | |
| --- | --- | --- | --- | --- | --- | --- | --- |
| **Province** | **Time period** | **Non-ICU to death** | **Non-ICU to recovery** | **Non-ICU to ICU** | **ICU to death** | **ICU to recovery** | **Post-ICU to recovery** |
| Eastern Cape | Wave 1 | 3 (1-8) [n=2,338] | 8 (5-12) [n=5,707] | 0 (0-5) [n=628] | 12 (3-33) [n=374] | 12 (7-21.25) [n=232] | 0 (0-1.25) [n=232] |
|  | Post-wave 1 | 3 (1-7) [n=360] | 8 (4-11) [n=1,426] | 0 (0-5) [n=138] | 10 (4-23) [n=61] | 10 (5.25-20) [n=70] | 0 (0-1) [n=70] |
|  | Wave 2 | 3 (1-7) [n=5,121] | 8 (5-11) [n=9,937] | 3 (0-7) [n=941] | 9 (4-16) [n=562] | 14 (7-23) [n=364] | 0 (0-0) [n=364] |
|  | Post-wave 2 | 5 (2-10) [n=223] | 9 (5-13) [n=790] | 0 (0-5) [n=112] | 10.5 (6-17.75) [n=46] | 8 (5-17) [n=65] | 0 (0-0) [n=65] |
|  | Wave 3 | 4 (2-8) [n=986] | 8 (5-11) [n=2,691] | 4 (0-7) [n=510] | 9 (6-15) [n=305] | 11.5 (5-20.25) [n=172] | 0 (0-1) [n=172] |
| Free State | Wave 1 | 4 (1-11) [n=1,167] | 8 (4-11) [n=5,404] | 0 (0-5) [n=557] | 13 (6-25) [n=295] | 7 (3-13) [n=235] | 3 (0-7) [n=235] |
|  | Post-wave 1 | 4 (1-9.5) [n=43] | 8 (4-11) [n=350] | 0 (0-3.25) [n=36] | 15 (10.5-19.75) [n=18] | 14 (6.5-19) [n=15] | 2 (0-6) [n=15] |
|  | Wave 2 | 4 (1-8) [n=737] | 8 (5-11) [n=2,582] | 3 (0-6) [n=324] | 9 (5-14) [n=205] | 8 (4-19) [n=105] | 3 (0-7) [n=105] |
|  | Post-wave 2 | 6 (2-10) [n=204] | 8 (5-11) [n=757] | 4 (0-7) [n=88] | 10 (4-16) [n=45] | 10 (4.5-19.5) [n=35] | 2 (0-9.5) [n=35] |
|  | Wave 3 | 4 (2-8) [n=1,395] | 8 (5-11) [n=5,000] | 3 (0-7) [n=747] | 7 (3-14) [n=432] | 6 (2-13) [n=249] | 5 (0-10) [n=249] |
| Gauteng | Wave 1 | 4 (2-11) [n=3,762] | 7 (4-12) [n=18,289] | 0 (0-4) [n=3,243] | 14 (5-29) [n=1,361] | 8 (4-16) [n=1,750] | 2 (0-6) [n=1,750] |
|  | Post-wave 1 | 5 (2-12) [n=582] | 6 (3-11) [n=4,236] | 0 (0-3) [n=889] | 10 (4-20.5) [n=247] | 7 (4-14) [n=609] | 0 (0-3) [n=609] |
|  | Wave 2 | 4 (2-9) [n=3,760] | 7 (4-11) [n=15,714] | 0 (0-5) [n=3,695] | 10 (5-17) [n=1,734] | 9 (5-15) [n=1,805] | 1 (0-5) [n=1,805] |
|  | Post-wave 2 | 6 (2-12) [n=1,000] | 7 (4-11) [n=5,050] | 0 (0-4) [n=1,304] | 12 (6-21) [n=451] | 7 (4-14) [n=777] | 0 (0-3) [n=777] |
|  | Wave 3 | 5 (2-10) [n=8,493] | 7 (4-11) [n=30,030] | 2 (0-6) [n=6,967] | 10 (5-17) [n=3,438] | 9 (4-16) [n=2,933] | 1 (0-5) [n=2,933] |
| KwaZulu-Natal | Wave 1 | 5 (2-14) [n=1,548] | 8 (5-12) [n=9,843] | 0 (0-5) [n=1,737] | 16 (7-34) [n=860] | 8 (4-16) [n=806] | 1 (0-6) [n=806] |
|  | Post-wave 1 | 5 (1-14) [n=294] | 7 (4-11) [n=2,554] | 0 (0-5) [n=320] | 10 (5-22.75) [n=122] | 9 (4-14) [n=169] | 1 (0-5) [n=169] |
|  | Wave 2 | 5 (2-10) [n=5,632] | 8 (5-12) [n=15,760] | 0 (0-7) [n=2,299] | 8 (4-16) [n=1,429] | 10 (5-18) [n=743] | 0 (0-5) [n=743] |
|  | Post-wave 2 | 7 (3-12) [n=498] | 8 (4-12) [n=2,781] | 0 (0-4) [n=347] | 9 (4-15.5) [n=155] | 7 (4-13) [n=165] | 0 (0-5) [n=165] |
|  | Wave 3 | 5 (2-9) [n=1,597] | 8 (5-11) [n=5,765] | 0 (0-5) [n=1,112] | 7 (3-14) [n=606] | 9 (5-15) [n=412] | 0 (0-4) [n=412] |
| Limpopo | Wave 1 | 4 (2-12) [n=329] | 6 (4-10) [n=1,605] | 0 (0-6) [n=164] | 11.5 (4.25-22.75) [n=86] | 7 (2.25-10.75) [n=62] | 6 (1-10.75) [n=62] |
|  | Post-wave 1 | 9 (2-19) [n=43] | 7 (4-10) [n=216] | 0 (0-3) [n=20] | 13.5 (4.25-24.25) [n=12] | 12.5 (4.5-20.5) [n=6] | 3 (0.5-4) [n=6] |
|  | Wave 2 | 3 (1-8) [n=1,700] | 6 (3-10) [n=3,196] | 0 (0-5) [n=253] | 7 (3-10) [n=179] | 6 (3-10) [n=56] | 4 (0-10.25) [n=56] |
|  | Post-wave 2 | 4 (1.25-10) [n=230] | 7 (4-11) [n=826] | 0 (0-5) [n=109] | 8.5 (4-13) [n=62] | 5 (2.5-9) [n=35] | 5 (2.5-9) [n=35] |
|  | Wave 3 | 4 (2-8) [n=1,437] | 6 (3-10) [n=3,634] | 3 (0-6.25) [n=340] | 7 (3-12) [n=207] | 4 (2-8) [n=95] | 5 (1-9) [n=95] |
| Mpumalanga | Wave 1 | 5 (1-20) [n=403] | 7 (4-11) [n=1,701] | 1 (0-7) [n=270] | 15 (5.5-28) [n=127] | 13 (4-25) [n=125] | 0 (0-6) [n=125] |
|  | Post-wave 1 | 5 (2-17.5) [n=107] | 6 (3-9) [n=724] | 0 (0-5) [n=101] | 11 (5-19) [n=45] | 11 (7.5-18.5) [n=51] | 0 (0-4.5) [n=51] |
|  | Wave 2 | 3 (1-7) [n=1,137] | 7 (4-11) [n=2,725] | 3 (0-7) [n=401] | 12 (5-18) [n=222] | 13 (5.5-23) [n=159] | 0 (0-5) [n=159] |
|  | Post-wave 2 | 4 (2-8) [n=457] | 7 (4-10) [n=1,819] | 3 (0-6) [n=324] | 12 (6-23) [n=164] | 11 (6-21.25) [n=140] | 0 (0-5) [n=140] |
|  | Wave 3 | 5 (2-9) [n=888] | 6 (4-10) [n=2,818] | 4 (0-8) [n=433] | 9.5 (4-17) [n=250] | 10 (4-19) [n=141] | 0 (0-5) [n=141] |
| Northern Cape | Wave 1 | 6 (2-14) [n=262] | 6 (3-10) [n=1,939] | 3 (0-8) [n=160] | 11 (4.5-21.5) [n=75] | 9 (5-25) [n=61] | 3 (0-8) [n=61] |
|  | Post-wave 1 | 5 (2.25-22.5) [n=22] | 5 (2.5-8.5) [n=107] | 4 (0.75-6) [n=8] | 10.5 (4.75-16.25) [n=4] | 8 (4.5-11.5) [n=3] | 5 (3-9) [n=3] |
|  | Wave 2 | 5 (2-12) [n=385] | 6 (3-10) [n=1,243] | 1 (0-6.25) [n=140] | 9 (5-15) [n=71] | 9 (4-24.5) [n=55] | 4 (0-10) [n=55] |
|  | Post-wave 2 | - [n=0] | - [n=0] | - [n=0] | - [n=0] | - [n=0] | - [n=0] |
|  | Wave 3 | 4 (2-9) [n=757] | 7 (3-11) [n=1,992] | 5 (0-9) [n=194] | 9 (4-17) [n=131] | 5.5 (3-19.25) [n=40] | 3 (0-7) [n=40] |
| North West | Wave 1 | 8 (2-18) [n=367] | 8 (5-13) [n=5,083] | 0 (0-3) [n=496] | 12 (5-27.5) [n=247] | 12 (6-17) [n=221] | 0 (0-3) [n=221] |
|  | Post-wave 1 | 8 (6-9) [n=11] | 9 (6-10) [n=324] | 0 (0-2) [n=9] | 4.5 (3.25-14) [n=6] | 6 (5-14.5) [n=3] | 1 (0.5-9) [n=3] |
|  | Wave 2 | 6 (2-12) [n=636] | 7 (5-10) [n=3,332] | 0 (0-6) [n=394] | 10 (4-16) [n=215] | 9 (5-15) [n=169] | 0 (0-3) [n=169] |
|  | Post-wave 2 | 6 (2-10) [n=354] | 8 (5-10) [n=1,949] | 0 (0-3) [n=252] | 8 (3.5-15) [n=107] | 8 (5-14) [n=103] | 0 (0-4) [n=103] |
|  | Wave 3 | 5 (2-9) [n=1,444] | 7 (4-10) [n=6,162] | 0 (0-6) [n=761] | 7 (3-13) [n=410] | 9 (4-16) [n=276] | 0 (0-3) [n=276] |
| Western Cape | Wave 1 | 6 (2-12) [n=2,450] | 7 (4-13) [n=12,047] | 0 (0-5) [n=1,809] | 15 (6-50) [n=675] | 9 (5-17) [n=994] | 1 (0-6) [n=994] |
|  | Post-wave 1 | 6 (2-12.25) [n=244] | 6 (3-11) [n=2,028] | 0 (0-5) [n=328] | 13 (5-26) [n=87] | 7 (4-14) [n=230] | 0 (0-4) [n=230] |
|  | Wave 2 | 6 (2-10) [n=4,419] | 7 (4-11) [n=16,378] | 2 (0-6) [n=2,114] | 9 (5-15) [n=959] | 8 (4-16) [n=1,056] | 0 (0-5) [n=1,056] |
|  | Post-wave 2 | 6 (2-11) [n=470] | 6 (2-11) [n=3,397] | 0 (0-6) [n=565] | 8 (5-15) [n=175] | 7 (3-15) [n=355] | 0 (0-5) [n=355] |
|  | Wave 3 | 5 (2-10) [n=3,306] | 6 (4-11) [n=10,992] | 3 (0-7) [n=2,014] | 9 (5-16) [n=933] | 8 (4-15) [n=881] | 1 (0-5) [n=881] |

**Figure A.** **Schematic of estimated probabilities and lengths of stay**

**
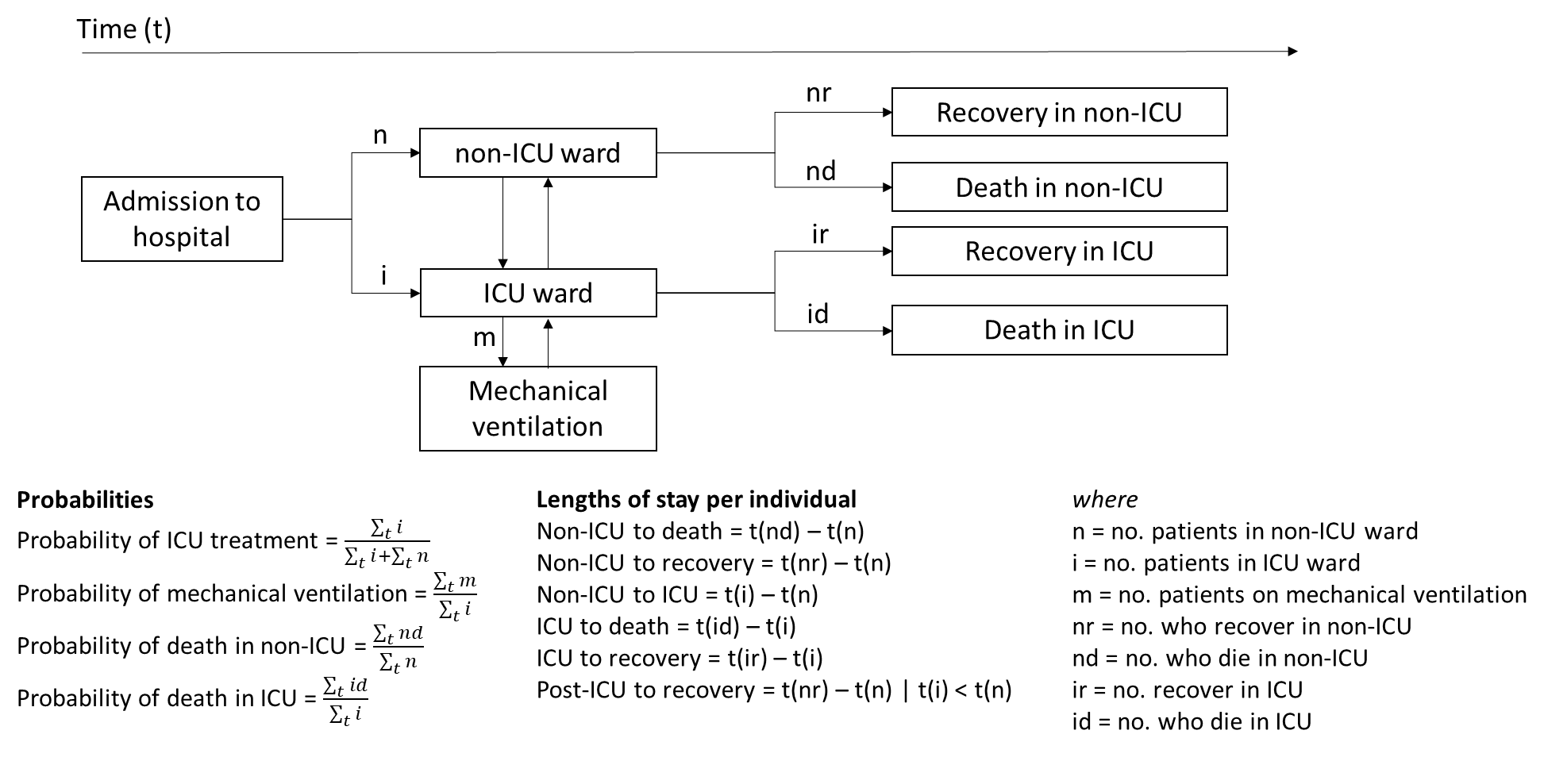
**

**Figure B. ICU admissions and hospital deaths over time including restrictions**^[[1]](#footnote-1)^


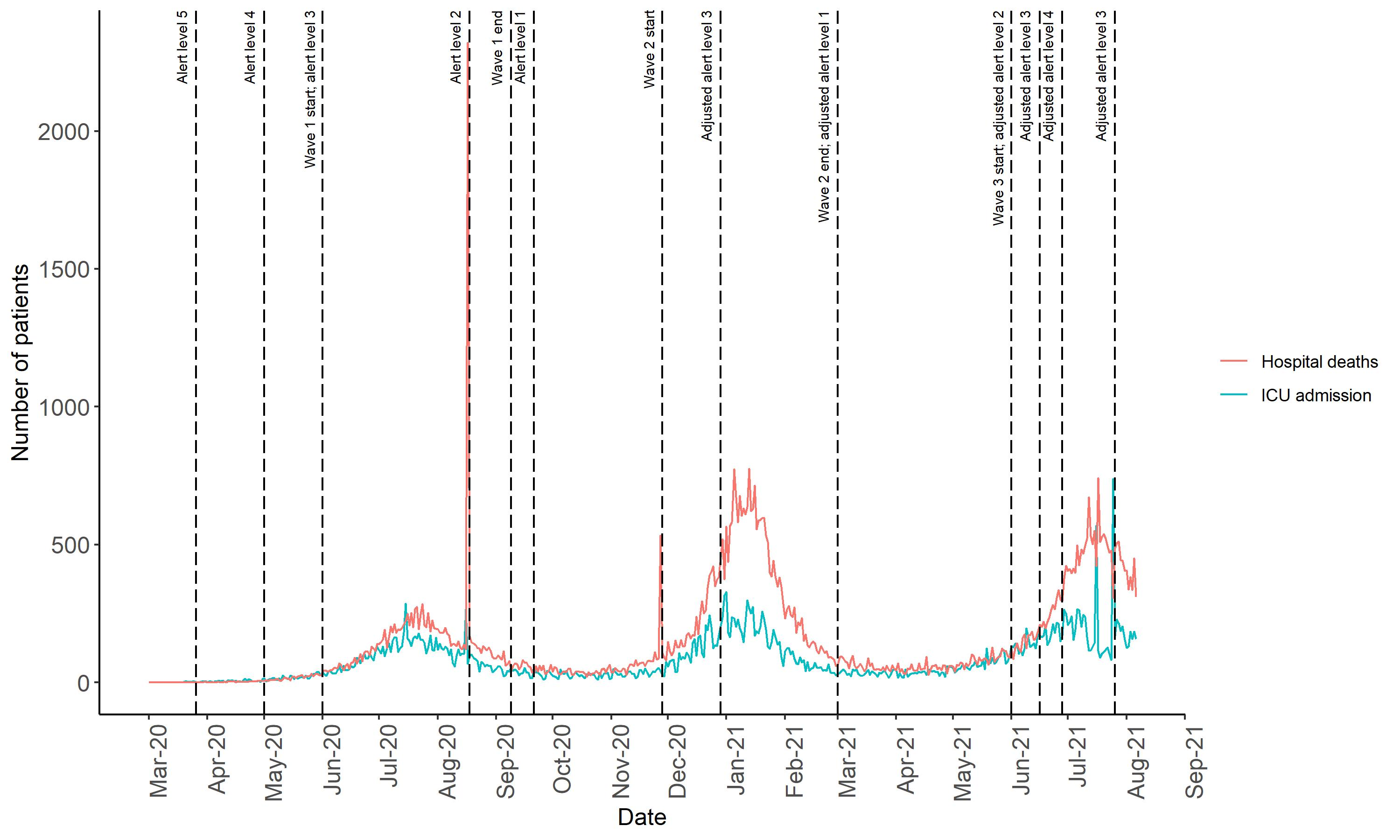


**Figure C. Flowchart depicting number of patients included for analysis**

**
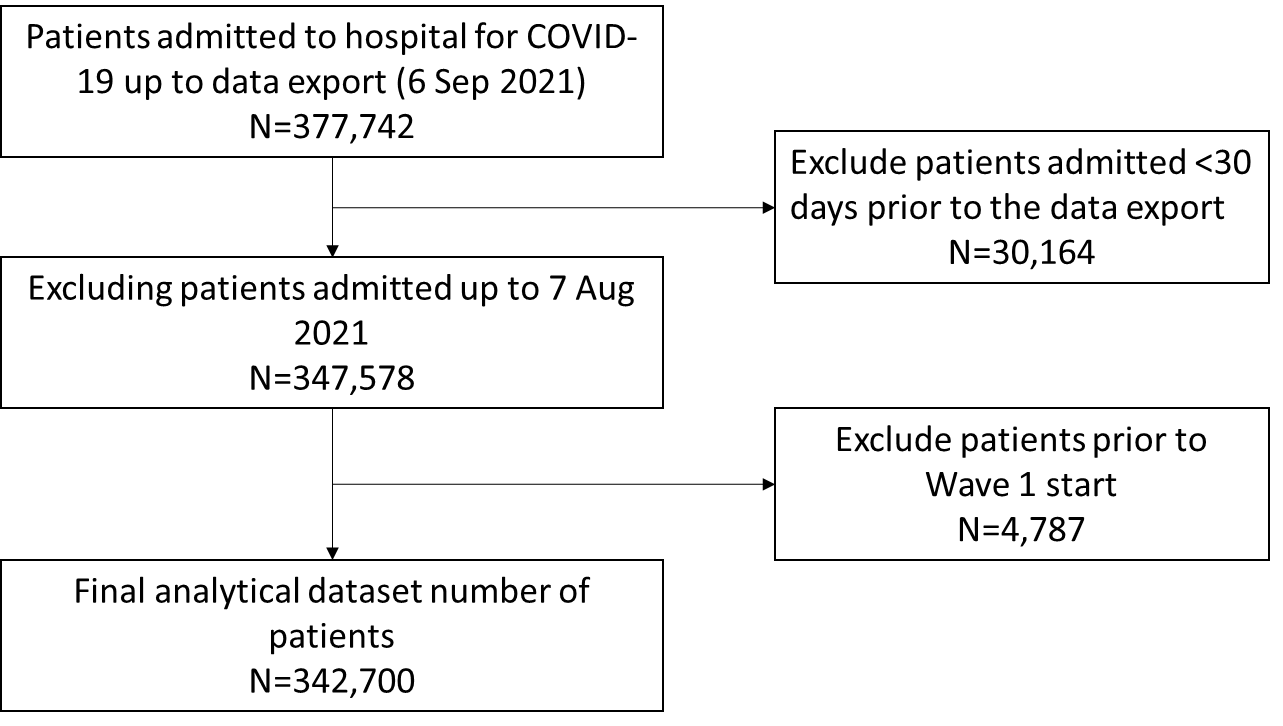
**

**Figure D. Length of hospital stay (75^th^ percentile, in days) in non-ICU and ICU to recovery or death, stratified by age, time period and public/private healthcare sector**


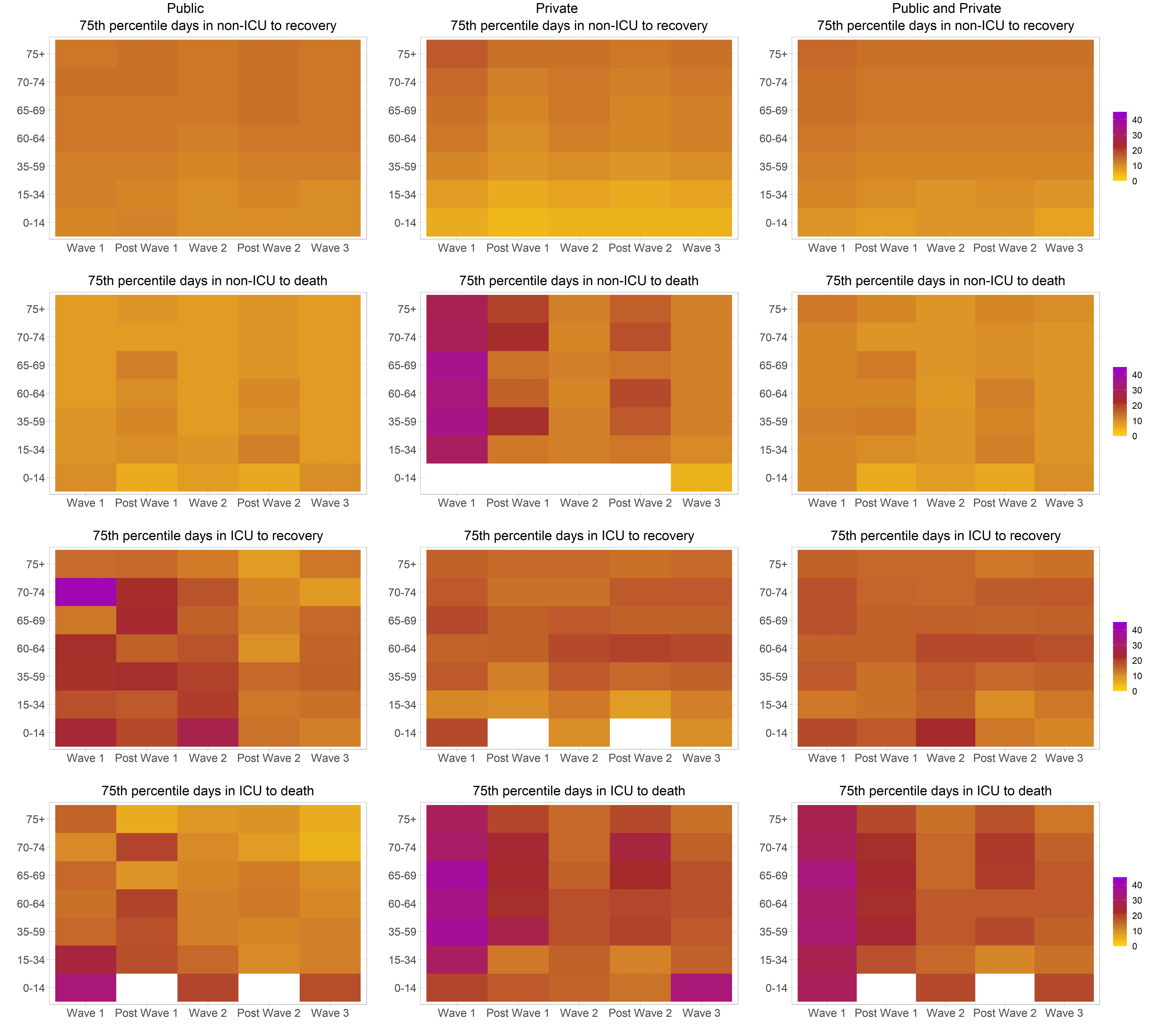


1. South African Government. Regulations and Guidelines - Coronavirus COVID-19. 2022.https://www.gov.za/covid-19/resources/regulations-and-guidelines-coronavirus-covid-19

   Alert Levels indicate the level of restrictions in place and services that were allowed to operate with Alert Level 5 being the strictest with only essential services allowed, public transport limited, no movement between provinces, to Alert Level 1 where most activities continued, with mask mandates. Adjusted alert levels are variations of the above-mentioned restrictions. [↑](#footnote-ref-1)
