# Supplementary material for: Differential in-hospital mortality and intensive care treatment over time: Informing hospital pathways for modelling COVID-19 in South Africa
Source: PLOS Glob Public Health. 2023 May 17;3(5):e0001073. doi: 10.1371/journal.pgph.0001073 (PMC10191304; doi:10.1371/journal.pgph.0001073)
Supplement: Attachment — Submitted filename: Responses to reviewers 09.02.2023.pdf [file pgph.0001073.s002.pdf]

Responses to reviewers

Journal Requirements:

1. We have noticed that you have uploaded Supporting Information files, but you have not included a list of legends. Please add a full list of legends for your Supporting Information files after the references list.

RESPONSE: We have added a list of legends for tables and figures in the Supporting information after the References.

2. Please amend your Data Availability Statement and indicate where the data may be found.

RESPONSE: We have included a Data Availability Statement in the Methods section

Additional Editor Comments (if provided):

I recommend the following additional minor edits to the manuscript

1. The authors should include a separate conclusion section to their manuscript where they outline key recommendations to policy makers in their country and the region as a whole.

RESPONSE: We have created a separate conclusions section and included recommendations to policy makers.

2. The authors have not done enough to outline key weaknesses and limitations to their study and approach.

RESPONSE: We have boosted our limitations section significantly with the following additions:

"Second, our data are censored at a time when the Delta wave (wave 3) had not yet subsided, and had just passed the peak in most provinces. Therefore, estimates of probability of death are possibly higher than they would have been if we had also been able to include the downturn of admissions during the end of the wave; this would also result in the probability of ICU admission being lower than expected for the Delta wave. There is also the possibility of underestimating lengths of stay and probabilities of death as censoring the data may exclude patients who would have had longer stays and likely poorer outcomes, however we found this was a small fraction as those still remaining in hospital in the last week of data censoring represented only 1% of all patients admitted during the Delta wave. Third, comorbidity data is likely self-reported, especially in public sector hospitals and/or dependant on the disease. In the case of it being self-reported, it may either be overestimated or underestimated dependant on the disease. Ideally one would run a quantitative bias analysis to try account for the self-report bias; however, that was beyond the scope of this paper. Fourth, we did not have data on SES; SES will influence risk of mortality, and though private/public sector might be a proxy, including SES may provide a more nuanced results and identify the most vulnerable patients within the health sector."

3. The paper will benefit by having a schematic which visual shows the methodology and what the analysis is trying to achieve

RESPONSE: We have created a schematic and included it in the supplementary material (Figure S1) with reference to it in the methods.

4. Some key covid vaccination citations for the country are missing and one would have expected to see them included

RESPONSE: Thank you for picking this up. We have updated to introduction to include citations to the Sisonke vaccination roll-out from Takuva et al and Bekker et al.

Reviewer #1:

Many thanks to the authors for their clearly presented and well-written manuscript. I have some minor comments and suggestions that I have detailed below, which I hope will help improve the overall quality of this paper.

I have only a few relatively major comments (which are still quite minor), just requesting additional clarity on what has been done in parts or further information to supplement what is presented:

1. Please clarify in the Methods regarding the source of the probabilities in the Results (paragraphs starting L172 and 188) — are these estimated from the log-binomial model also, or just calculated directly from the observed data? If estimated, please include CI's. This makes it slightly unclear which analysis is being referred to by "In an age-stratified analysis…". Please also note that you switch from $0 \leq p \leq 1$ to $0 \leq p \leq 100\%$ between the two paragraphs.

RESPONSE: We have amended the Methods section to clarify the two different approaches to this data with the following sentence: "We estimated probabilities of ICU admission, mechanical ventilation and death in non-ICU and ICU across all wave periods based on observed data." **Following this, there are methods describing the use of the log-binomial model:** *"We fitted a log-binomial model to the data to estimate relative risk of ICU admission and death in ICU/non-ICU, comparing 'wave periods'…"* **The switch between $0 \leq p \leq 1$ to $0 \leq p \leq 100\%$ between the two paragraphs is due to us moving between describing results from estimating probabilities to describing results from the log-binomial model.**

2. On Lines 182–187, you describe the possible reduction in deaths had the probability of death in non-ICU and ICU been the same during waves as it was between waves. It would be helpful to describe a little further/more precisely how this was calculated in the methods, e.g., is it a single, baseline estimate that is then modified by the RR associated with the different waves/variants to obtain different probabilities for each period, or similar? Noting that you exclude some of your population during the Delta outbreak at the end of your study period from the analysis, it is possible that your estimates of the probability of death during this period are not accurate, and it would be helpful to expand on this in the Discussion.

RESPONSE: We have included a sentence in the methods section to describe this calculation: *"We estimate the number of deaths that could have been avoided if the healthcare system had not been overburdened during the wave periods, by applying the age-, sector- and ward-specific between-wave probabilities of death to the number of patients admitted during a wave period."*
Regarding the Delta outbreak, this wave started around April – May 2021 in South Africa and though we do not capture the full period of the Delta wave beyond August, we do evaluate data during the wave's peak. We have however added to the Discussion the following sentence to limitations: *"Secondly, our data are censored at a time when the Delta wave (wave 3) had not yet subsided, and had just passed the peak in most provinces. Therefore, estimates of probability of death are possibly higher than they would have been if we had also been able to include the*

downturn of admissions during the end of the wave; this would also result in the probability of ICU admission being lower than expected for the Delta wave."

3. Could you please define in the Methods whether your "ICU length of stay" is the time spent in ICU, or the total hospital length of stay for individuals who spent time in the ICU at some point during their hospital stay. On a related note, is it possible with your data to include finer resolution on the length of stay and pathways through the hospital — e.g., can you also include estimates of the median (IQR) length of stay for individuals who were admitted to non-ICU beds before progressing to ICU beds? Similarly, are there data on length of stay post ICU, in a non-ICU bed, before discharge? These kind of estimates are helpful for clinical forecasting of hospital occupancy.

RESPONSE: ICU length of stay is the time spent in ICU, and not the length of the total hospital stay for patients who spent some time in ICU. We do have estimates for the time spent in non-ICU prior to ICU admission, and time spent in non-ICU after ICU discharge (post-ICU in non-ICU) and have included them in the supplementary material. We have clarified this in the methods with the following sentence: *"We estimated length of stay in non-ICU wards (for those never enrolled into ICU) and in ICU from admission into the ward type to recovery/discharge or death; we further estimate the time spent in non-ICU prior to ICU transfer (non-ICU to ICU) and time in non-ICU after ICU discharge (post-ICU to recovery)."*
Then added to the results: *"Time in non-ICU prior to ICU admission, and time in non-ICU after ICU discharge was generally very short across all waves and age groups (Table S7)."*

4. I also would suggest that, in addition to Figure 2, you include a figure that shows a greater percentile (e.g., 75% or 90% – noting that 75% is presented in Table S3 as the upper IQR value), to give some visual indication of the variability in length of stay? This could be included in as a supplemental figure.
RESPONSE: We have included the suggested figure into the supplemental material *"Figure S2: Length of hospital stay (75th percentile, in days) in non-ICU and ICU to recovery or death, stratified by age, time period and public/private healthcare sector"* **and make reference to it in the Results.**

Minor comments and typographical errors:

Abstract, Methods: The percentages and aRR's are swapped for death in non-ICU and ICU.
RESPONSE: Thank you, we have corrected this.

L66: Missing space between "2020which".
RESPONSE: Thank you, we have corrected this.

L68: Capitalise 'delta'.
RESPONSE: We have corrected this.

L71: To provide context to the 320,000 individuals that had been vaccinated, could you please provide the population of South Africa (or perhaps size of 12+ population), here or earlier in the Introduction.

RESPONSE: We have amended the mentioned sentence to add.. *"…, a small portion compared to the 43 million South Africans >15 years"* **– unfortunately we are limited to using the same age groups as described in government statistics reports.**

L102: Could you please clarify what exactly is meant by "the response of healthcare professionals"? Is this referring to the changing standard-of-care throughout this period?
RESPONSE: Yes, this is what we were referring to. We have amended this sentence to *"As the COVID-19 pandemic progressed, the standard-of-care treatment as well as the healthcare-seeking behaviour of individuals changed in response to the limited available capacity."*

L106: Missing "during" between "estimated the".
RESPONSE: Thank you for picking this up. We have corrected it.

L107–108: Is "…in mortality and treatment to ICU care…" meant to say "…in mortality and progression to ICU care…"?
RESPONSE: Thank you. This sentence has been amended to *"assess the impact waves could have played in mortality and admission to ICU care".*

L112: Missing the word "in" at the end of this line (i.e., "…hospitalisations in South Africa…").
RESPONSE: Thank you. We have corrected it.

L117-119: You describe your data processing such that you allowed sufficient time for patients to experience an outcome. Could you please describe how many individuals this removed from your analysis with this processing step? I presume it is a very small fraction, but this would likely result in an underestimate of some of your outcomes (especially LoS) for those that had longer stays and potentially then worse outcomes, as related to the Delta wave.
RESPONSE: We have created a flow diagram depicting the number of individuals whose data we extracted, how many were excluded, and the number included in the final dataset, and included this figure in the supplementary material, while referencing it in the Results. As for how the censoring may have led to underestimated LoS and probabilities of death for those in the Delta wave, there were approximately 1,000 patients remaining in hospital in the last observed week of the data, representing ~1% of the >100,000 patients admitted during the Delta wave. We have included this in our Discussion: *"There is also the possibility of underestimating lengths of stay and probabilities of death as data censoring may have excluded patients who would have had longer stays and likely poorer outcomes. However, we found this was a small fraction as those still remaining in hospital in the last week of data censoring represented only 1% of all patients admitted during the Delta wave."*

L152–154: The statement about mechanical ventilation trends here is far less strong than the summary statement provided in the abstract. Perhaps the abstract requires slightly more caveats on this result? It would also be helpful to present the estimates (and CIs) in text as are done for other aRR's.
RESPONSE: We have amended the abstract to read *"Mechanical ventilation was more likely to occur during a wave overall (aRR 1.18 [1.13-1.23]), but patterns between waves were inconsistent"* **and in the main text we have amended the sentence to include estimates and CIs:** *"Patterns of treatment*

with mechanical ventilation were less clear, but overall there was more treatment with mechanical ventilation during wave periods compared to between-wave periods (adjusted risk ratio (aRR) 1.18 [95% confidence interval (CI) 1.13-1.23]."

L162: On the statement "…, likely due to lower ICU capacity available in the public sector". Are there data on the number of ICU beds by sector that you could couple with the percentage of the population that have private health access, to support this statement? Appreciate that these may not be easy numbers to obtain and so not feasible.
RESPONSE: Unfortunately, there is no reliable data available on the number of ICU beds by sector for South Africa. In addition to this, hospitals and districts were continuously adding and removing bed capacity for COVID-19 patients as the pandemic progressed and as the needs of the population changed. So while we have an overall idea of bed capacity, i.e. less beds available in the public than in the private sector in which majority of the population are serviced, the use of these figures as a metric to inform our analysis was not possible.

L189: "during a waves period" should be "during a wave period".
RESPONSE: We have corrected this wording.

L197: "risk of death in ICU death decreased" should be "risk of death in ICU decreased".
RESPONSE: Thank you, we have corrected this wording.

Paragraph starting line 205: spaces are missing between "to" and number throughout, e.g., "7 to10 days".
RESPONSE: Thank you, we have added the missing space.

L212: The patterns in Fig 1 suggest that the probability of mechanical ventilation increased over time (on average), across age groups. While it is very plausible just due to changing standard-of-care over time, is it possible that there were also fewer ventilators (and appropriately trained staff to operate them) available during the earlier stages of the pandemic? Is there information on ventilators purchased by the government during 2020?
RESPONSE: Unfortunately this data is not available to incorporate into this analysis. We have included a sentence in the Discussion: *"We did not see as consistent a pattern of mechanical ventilation use with wave and between-wave periods as with mortality, and this may likely be due to a combination of increased ventilation capacity as more ventilators were acquired as well as a change in the use of ventilation as new therapeutics were introduced later on in the pandemic."*

Finally, I appreciate that these data may be challenging to access and so this may not be feasible, but is there any information available on hospitals reaching capacity during any of the waves that could support statements about ICU admissions, death etc. changing as capacity are reached? E.g., just a number/proportion of hospitals that reached their "effective bed" (i.e., beds and staff) capacity during each wave?
RESPONSE: Bed capacity data across hospitals was not reliable enough to be able to determine effective bed use.

Reviewer #2:

Thank you for giving me the opportunity to review this important manuscript. I appreciate the author's effort in doing this research. I have a list of suggestions/comments, which are listed below.

Introduction:

1. In paragraph 3, the authors discuss current research in low- and middle-income nations. Though there is limited research in these countries, it would be helpful for readers if the author could search for more recent research on low- and middle-income countries and include it here.

RESPONSE: The majority of research conducted were in cohorts early on in the pandemic, pre-2021. We revised our literature review, searching PubMed for any publications reporting research on COVID-19 hospitalization in LMIC and found 660. Of these, most were excluded by publication title due to being irrelevant. Forty-eight papers were relevant, but most reported on data collected on the initial wave in 2020 only. We include a few additional citations which were most relevant to our manuscript.

We have therefore amended the sentence in the Introduction to say *"There have been limited published data from low- and middle-income countries (LMIC) describing COVID-19-related hospitalisation and inpatient care, especially beyond the first wave of COVID-19 in 2020."* **and have added on to the existing literature review with the following** *"Solanki et al (14) report on the private sector experience in South Africa, and also found an increased risk of hospitalisation and mortality amongst those with comorbidities and older age groups, and a variable risk of mortality between provinces. A review of studies reporting lengths of stay across multiple countries found only a single study in Egypt, and it had comparatively shorter length of stay compared to other countries (15). More recent studies focused on the fourth wave, dominated by the less severe Omicron variant (16–20)."*

2. It would also be useful to briefly describe research conducted in middle- and high-income countries, as this would help readers understand what is known and where gaps exist from a global perspective.

RESPONSE: While we agree this would be useful for the reader who is interested in middle- and high-income countries, this is beyond the scope of this paper as our focus is on low- and middle-income countries.

Methods:

1. A data extraction flowchart would be helpful for readers.

RESPONSE: We have created a flow chart depicting the number of patients extracted and numbers excluded from analysis, and added it to the supplementary material (Figure S1).

2. "Patients with at least one of the following: hypertension, diabetes, cardiac disease, chronic pulmonary disease, asthma, chronic renal disease, any malignancy, past or active tuberculosis, HIV infection or obese, were classified as having any comorbidity." Self-reported? Self-reporting of diagnoses is a common way to collect health data, and patient-reported comorbidities are reliable, especially for chronic conditions (e.g., diabetes). Depending on the disease and other factors, patients' reports may underestimate or overestimate relevant comorbidities. Was an attempt made to account for bias in this study? This would be helpful to include in this paragraph.

RESPONSE: Comorbidities were either self-reported or captured from patient medical records – we have clarified this in the methods. Formally accounting for the bias through methods such as quantitative bias analysis is beyond the scope of our analysis, and as such we have included this as a limitation in the Discussion: *"Third, comorbidity data is self-reported for some patients, especially in public sector hospitals and/or for specific diseases. This may lead to us having either overestimated or underestimated co-prevalence. A quantitative bias analysis accounting for this self-report bias is however, beyond the scope of this paper."*

3. A binary variable called comorbidity has been created and used in this study. Have the authors investigated whether patients with a single condition (e.g., diabetes or asthma) had a higher risk of ICU admission or mortality? In addition to the comorbidity variable, I would recommend performing analysis with a specific condition in the model because not all conditions have the same degree of influence on ICU admission and mortality.
RESPONSE: We have added a separate analysis where we include single conditions in the model (supplementary material, Table S5). We have also summarised the findings in the results: *"Most comorbidities increased risk for ICU treatment, in particular patients with diabetes were 24% more likely to be admitted to ICU (aRR 1.24 [1.20-1.27]) (Table S5). Similarly, the presence of most comorbidities led to increased risk of non-ICU mortality, with diabetes (aRR 1.25 [1.22-1.28]), chronic renal failure (aRR 1.20 [1.15-1.26]), malignancy (aRR 1.29 [1.16-1.43]), tuberculosis (aRR 1.21 [1.10-1.31]) and HIV (aRR 1.26 [1.21-1.31]) being particularly strong risk factors; with a similar pattern of comorbidities being associated with an increased risk of ICU mortality (Table S5)."*

4. In Table 1, please include statistics on specific conditions (hypertension, diabetes, cardiac disease, chronic pulmonary disease, asthma, chronic renal disease, any malignancy, past or active tuberculosis, HIV infection, or obesity).
RESPONSE: We have updated Table 1 to include statistics of individual comorbidities, in addition to our aggregate variable for "Any comorbidity".

5. It would be beneficial to provide justification for the log-binomial model. Despite its benefits, there are disadvantages (e.g., boundedness of parameter space). The reader would benefit from a brief explanation of why log-binomial models were chosen over other models (such as logistic regression) for this study.
RESPONSE: We have added the following sentence to the methods: *"A log-binomial model was chosen as it provides unbiased estimates of the adjusted relative risk and avoids overestimation in cases where the outcomes modelled is common as is the case for logistic regression."*

6. The model description paragraph needs to include information about model development steps. This study took two steps. This study first considered model outcome and wave period while adjusting for age, sex, comorbidities, province, and public/private sector. This study considered stratified analysis by public/private sector in the second step.
RESPONSE: The first step is described in the methods, however, we added a sentence in the methods to describe the second step with *"We conducted a stratified analysis by public/private sector as the sectors differ widely in available resources and, as a result, the quality of the services they are able to provide."*

7. Before conducting stratified analysis, has this study investigated the interaction between "wave period" and "public/private sector"?
RESPONSE: We conducted this analysis and found significant interactions for most outcomes: "ICU treatment", "mechanical ventilation" and "non-ICU death", but not "ICU death"- the latter likely not significant due to the large sample size required for interaction terms. Another (and the main) reason for conducting a stratified analysis for public/private sector was due to these sectors being so vastly different with respect to resources available and quality of service as a result. We have included this explanation in the Methods (see response to comment (6) above). To address the results of the interaction term, we have included the following sentence in the Results section: *"There were significant interactions between wave period and public/private sector when evaluating the outcomes ICU treatment, mechanical ventilation and death in non-ICU, but not for death in ICU (Table S4)."*

8. Has this research examined the relationship between "public/private sector" and "province"? It is essential because the number of hospitals can vary by province.
RESPONSE: We have now included an interaction term between public/private sector and province and included the results in the supplementary material. We have updated the results with the following sentence: *"Interactions between province and public/private sector were mostly significant across all outcomes modelled (Table S4)."*

9. This model building paragraph should also contain information about what was done with the "Unknown" category of the comorbidity variable during development of model.
RESPONSE: We have included the following sentence in the methods: *"Observations with missing data were excluded from the models; the reporting of comorbidities was the variable most affected by missing data, with 21% of the patient population having "unknown" comorbidity status."*

Results:
1. Having trend graphs for all of the most important outcome variables will help visualise the trend of ICU admission and mortality. On the graph, it would also be useful to highlight COVID waves and restrictions period.
RESPONSE: We have created a line graph depicting ICU admission and mortality over time with restriction periods and wave start dates highlighted to give the reader an idea of how the pandemic progressed nationally. This was included in the supplementary material (Figure S2) and reference was made to it in the Results.

2. The result section can be improved further by including information about the additional analysis suggested in the methods section's comments.
RESPONSE: We have addressed these additional analyses (see previous responses)

3. The paragraph "Patterns of hospital lengths of stay in non-ICU and ICU across waves" may include statistics on how length of stay differed by hospital type (i.e., public versus private) and provinces.
RESPONSE: We have included tables for these results in the supplementary material and added the following sentence in the Results *"Across all age groups, private sector had longer lengths of stay for those who died in ICU and non-ICU compared to public sector, while time to recovery in ICU*

and non-ICU was longer in public than private sector (Table S8). There was some variability in lengths of stay between provinces across all metrics, but no distinguishable pattern emerged (Table S9)."

Discussion:
1. In paragraph 4, there is a discussion about the type of hospital. This paragraph can be improved by including a discussion of hospital accessibility and its potential impact on the outcome. Patients from different socioeconomic backgrounds are likely to have varying levels of access to hospitals, which may influence ICU admission and mortality.
RESPONSE: We have added the following to the Discussion: *"In addition, access to treatment is often modified by socioeconomic status (SES), with those with lower SES often having less access to hospital care than those with higher SES. This could explain the underrepresentation of public sector patients in this data. Multiple factors come into play when we consider how including SES would have affected our key parameters: lower SES could have worsened outcomes due to poorer health, higher rates of comorbidities, or an increased burden on the healthcare system that is already at capacity; or it could not have had any impact as the latter effects are removed after access to care improves their outcomes."*

2. The limitation section also needs to highlight the limitations of using self-reported comorbidity and not having data on socioeconomic status at the individual level and residential area level. Socioeconomic status is likely to influence various health conditions, hospital service access, and utilisation. I understand that these data may not be available but acknowledging them would be beneficial for future research as well as interpretation of current research findings.
RESPONSE: We have included the following in the limitations: *"Third, comorbidity data is self-reported for some patients, especially in public sector hospitals and/or for specific diseases. This may lead to us having either overestimated or underestimated co-prevalence. A quantitative bias analysis accounting for this self-report bias is however, beyond the scope of this paper. Fourth, we did not have data on SES; SES will influence risk of mortality, and though private/public sector might be a proxy, including SES may provide a more nuanced results and identify the most vulnerable patients within the health sector."*

3. Policy recommendations can be strengthened further by providing information on multisectoral approaches to strengthening health systems.
RESPONSE: We have added the following sentence to the conclusion section: *"This work can, along with modelling projections and scenarios, support the work of policy makers and COVID-19 hospital readiness or management teams in understanding the implications of constrained hospital capacity on in-hospital deaths, and allow them to, within limits, boost available capacity and put in place agile processes to upscale and downscale services within pandemic waves. Further, the South African government and governments from other LMIC should engage in multisectoral activities to strengthen its health systems as part of the next steps towards pandemic preparedness."*
